# Adulteration prevention-oriented systematic review of chemical constituents in the genus *Rhodiola* and strategic outlook for species identification based on chemical constituents

**DOI:** 10.3389/fphar.2026.1872755

**Published:** 2026-08-12

**Authors:** Jiahui Li, Xiaobo Sun, Geng Li

**Affiliations:** 1 Institute of Medicinal Plant Development, Chinese Academy of Medical Sciences and Peking Union Medical College, Beijing, China; 2 Institute of Chinese Materia Medica, Chinese Academy of Traditional Chinese Medicine, Beijing, China

**Keywords:** adulteration, chemical constituents, chemical markers, *Rhodiola*, species identification

## Abstract

*Rhodiola* species are renowned medicinal plants with centuries of traditional use and broad pharmacological effects. In recent years, market and economic factors have fueled widespread adulteration in the global *Rhodiola* product market. More critically, much potential adulteration may remain undetected, since authenticating the species of raw materials in processed products is particularly challenging: morphological, microscopic, and genetic methods are often invalidated by manufacturing processes. In contrast, phytochemical constituents remain traceable from raw materials to finished products, offering a promising avenue for authentication. Regrettably, the lack of reliable chemical markers for *Rhodiola* species identification hinders effective adulteration detection and market supervision. This paper aims to comprehensively review phytochemical constituents from the genus *Rhodiola* as the critical first step toward marker discovery. Multiple academic databases were searched to summarize chemical names, molecular formulas, structures, and botanical sources of documented compounds. The genus exhibits remarkable chemical diversity, with over 500 compounds classified into eight groups: phenylalkanoid derivatives, phenylpropanoids, flavonoids, terpenoids, phenolics and organic acids, cyanogenic glycosides, steroids, and others. Further analysis qualitatively delineates both interspecific chemical similarities (most compounds are shared across species) and differences (distinct combinations of shared compounds and compounds currently known from single species). These findings collectively elucidate the inherent challenges and potential feasibility of discovering chemical markers for *Rhodiola* species identification. Meanwhile, the consolidated phytochemical data serves as a foundational compound database, ensuring higher accuracy and reliability in subsequent UPLC-MS-based compound identification. Additionally, to reduce analytical complexity in marker exploration, this paper advocates shifting the focus from solely species-specific compounds to broader chemical characteristics (encompassing species-specific compounds, content levels, and content ratios), and proposes a hierarchical “chemical characteristics + classification tree” framework for stepwise identification. Finally, we outline a research plan to implement these strategies, comprising the completed phytochemistry review, sample collection, UPLC-MS-based qualitative profiling, UPLC-based quantitative analysis, and multivariate data analysis. In conclusion, this paper highlights the importance of developing a reliable chemical identification method for *Rhodiola* species, provides an essential chemical foundation and strategic guidance for future marker discovery, and ultimately aims to solve raw material authentication in *Rhodiola* preparations to combat adulteration and strengthen market supervision.

## 1 Introduction

*Rhodiola* species (Crassulaceae) are highly valuable medicinal plants predominantly native to Asia, Europe, and North America. For centuries, their roots and rhizomes have been integral to traditional medicine, and are now recognized for multifaceted pharmacological effects, including alleviating fatigue (Wang et al., 2024), delaying aging (Liang et al., 2023), preventing high-altitude sickness (Ma et al., 2022), improving cardiovascular function (Chen et al., 2022), treating neurodegenerative disorders (Zhang et al., 2019), combating cancer (Zhang et al., 2020), mitigating stress (Ivanova Stojcheva and Quintela, 2022), and boosting immunity (Skopńska-Rózewska et al., 2008).

The applications of *Rhodiola* species exhibit marked regional diversity, with *R. crenulata* and *R. rosea* being the two most extensively studied and widely utilized species. *R. crenulata*, primarily used in China, holds a unique status as the only species officially listed in the Chinese Pharmacopoeia (Commission, 2020). Its therapeutic value has been validated through over a millennium of clinical practice, supported by numerous traditional prescriptions and Chinese patent medicines. In contrast, *R. rosea* dominates commercial trade in Europe and North America. Officially recognized in the pharmacopoeias of Russia and the United States (Booker et al., 2016a), its extracts are widely marketed as dietary supplements in many countries to meet various healthcare needs. Additionally, more than 20 other species within the genus *Rhodiola* have been reported to possess medicinal properties. Among these, at least 10 species—including *R. kirilowii*, *R. fastigiata*, *R. dumulosa*, *R. yunnanensis*, and *R. sachalinensis*—are commercially available in herbal medicine markets as raw materials or decoction pieces (Booker et al., 2016a).

It is noteworthy that accumulating studies have highlighted the significant variations in pharmacological profiles among different *Rhodiola* species, such as differences in antioxidant and anti-fatigue capacities between *R. crenulata* and *R. rosea* (Dong et al., 2020; Abidov et al., 2003), as well as anti-hypoxia activity between *R. tangutica* and *R. kirilowii* (Chen et al., 2010). These results remind us that although medicinal *Rhodiola* species share similar therapeutic properties, the substitution or mixing of raw materials from different species may pose a potential risk to the consistency and stability of clinical efficacy. Unfortunately, this risk has been exacerbated by growing market demand and considerable economic benefits in recent years, leading to serious adulteration issues in *Rhodiola* products. Given the wide array of *Rhodiola* species available commercially, such adulteration is predominantly characterized by the substitution or mixing of raw materials in species-specific *Rhodiola* preparations with other congeners. Booker et al. assessed the authenticity of 39 commercial *Rhodiola* products sourced from various UK suppliers (Booker et al., 2016b). The absence of rosavin (a key marker compound for *R. rosea*) but presence of salidroside (an active constituent in nearly all *Rhodiola* species) in five samples claiming to contain *R. rosea* indicated the substitution with other *Rhodiola* species. Two samples showed no detectable levels of either rosavin or salidroside, suggesting possible adulteration with non-*Rhodiola* species. Around 80% of the remaining samples displayed lower rosavin content than registered products, implying possible adulteration with other *Rhodiola* species or the use of poor-quality raw materials. Similarly, Ma et al. examined 18 commercial products labeled as *R. rosea* root powder extract from eight suppliers (Ma et al., 2011). Their findings showed that one-third of the products contained no rosavins (a collective term for rosavin, rosarin, and rosin), and five others failed to meet label claims regarding rosavins or salidroside content. Another authenticity investigation into seven commercial *R. rosea* products sold online in Austria and Germany (in tablet or capsule form) revealed that one product contained neither rosavins nor salidroside, while another was missing rosin and salidroside (Langeder and Grienke, 2021). Analogous adulteration cases for *R. rosea* preparations have also been reported in Poland (Nikolaichuk et al., 2022), Ukraine (Khokhlova and Zdoryk, 2020), and Bulgaria (Marchev et al., 2020). These findings collectively underscore the global prevalence of adulteration issues in *Rhodiola* products.

Another noteworthy issue is that, although a wide range of commercial preparations derived from various *Rhodiola* species (e.g., *R. crenulata*, *R. rosea*, *R. wallichiana*, and *R. kirilowii*) are available on the market—many of which clearly specify the source species of their raw materials on product labels (Fan et al., 2019)—nearly all reported adulteration cases involve *R. rosea* preparations, as determined by the absence or substandard levels of rosavins. It does not necessarily mean that adulteration is absent in preparations from other *Rhodiola* species. This phenomenon can be largely attributed to the limitations of existing species identification approaches for *Rhodiola*, which cannot be effectively applied to authenticate raw materials in non-*R. rosea* preparations, rendering adulteration in these products undetectable.

The frequent occurrence of adulteration and the substantial risk of undetected adulteration in *Rhodiola* products necessitate the urgent strengthening of market supervision. Developing a more efficient species authentication method—especially one applicable to commercial *Rhodiola* preparations—would be pivotal in addressing this pressing problem. However, identifying the species of raw materials in herbal preparations is particularly challenging, as the morphological, microscopic, and genetic characteristics traditionally employed for species identification are often degraded or destroyed during manufacturing processes. In contrast, phytochemical constituents retain their traceability throughout the entire manufacturing process—from raw materials to finished products. This is why chemical markers have been consistently adopted as reliable indicators for detecting adulteration in *R. rosea* preparations across the aforementioned studies. Regrettably, for *Rhodiola* species other than *R. rosea*, no chemical markers capable of effectively distinguishing a target species from its congeners have yet been discovered. Therefore, future research should focus on discovering chemical markers that can reliably distinguish *Rhodiola* species.

In recent years, advances in analytical techniques have greatly facilitated the in-depth exploration of *Rhodiola* species, with chemical analysis remaining a central research focus. Multiple studies using ultra-high performance liquid chromatography (UPLC), high performance liquid chromatography (HPLC), nuclear magnetic resonance (NMR), and high performance thin layer chromatography (HPTLC), coupled with multivariate data analysis, have revealed significant variations in chemical constituents among different *Rhodiola* species (Booker et al., 2016a; Ma et al., 2021; Liu et al., 2013; Li et al., 2019). Liu et al. further demonstrated a high degree of consistency between chemotaxonomic classification and genetic classification of *Rhodiola* species (Liu et al., 2013). These species-dependent variations offer robust scientific evidence for identifying *Rhodiola* species based on their chemical constituents.

Considering the pressing need to address adulteration issues and the viable potential of discovering chemical markers for identifying *Rhodiola* species, this paper comprehensively reviews the chemical constituents isolated and identified from the genus *Rhodiola* over the past few decades. As the first step toward marker discovery, this review clarifies the current research status regarding the chemical constituents of various *Rhodiola* species and functions as an indispensable compound database for the next research phase. In doing so, this work lays a crucial chemical foundation for supporting subsequent research on marker discovery. Building on this foundation, in direct response to the significant difficulty in identifying *Rhodiola* species posed by chemical complexity, we further propose a series of targeted insights and strategies to guide a more effective exploration of chemical markers. Finally, we outline a future research plan that emphasizes key investigative directions and experimental priorities to advance our research endeavors.

The full scientific names and corresponding abbreviations of *Rhodiola* species involved in this paper are listed in Table 1.

**TABLE 1:** Full scientific names and abbreviations of *Rhodiola* species involved in this paper.

Full scientific names	Abbreviations
*Rhodiola crenulata* (Hook. f. & Thoms.) H. Ohba	*R*. *crenulata*
*Rhodiola rosea* L.	*R*. *rosea*
*Rhodiola kirilowii* (Regel) Maxim.	*R*. *kirilowii*
*Rhodiola fastigiata* (Hook. f. & Thomson) S. H. Fu	*R*. *fastigiata*
*Rhodiola wallichiana* var. *cholaensis* (Praeger) S. H. Fu	*R*. *wallichiana*
*Rhodiola tangutica* (Maxim.) S. H. Fu	*R*. *tangutica*
*Rhodiola sachalinensis* Boriss.	*R*. *sachalinensis*
*Rhodiola yunnanensis* (Franch.) S. H. Fu	*R*. *yunnanensis*
*Rhodiola dumulosa* (Franch.) S. H. Fu	*R*. *dumulosa*
*Rhodiola quadrifida* (Pall.) Fisch. & C. A. Mey.	*R*. *quadrifida*
*Rhodiola sacra* (Prain ex Raym.-Hamet) S. H. Fu	*R*. *sacra*
*Rhodiola bupleuroides* (Wall. ex Hook. f. & Thomson) S. H. Fu	*R*. *bupleuroides*
*Rhodiola henryi* (Diels) S. H. Fu	*R*. *henryi*
*Rhodiola himalensis* (D. Don) S. H. Fu	*R*. *himalensis*
*Rhodiola tibetica* (Hook. f. & Thomson) S. H. Fu	*R*. *tibetica*
*Rhodiola heterodonta* (Hook. f. & Thomson) Boriss.	*R*. *heterodonta*
*Rhodiola gelida* Schrenk., Fischer & C. A. Meyer	*R*. *gelida*
*Rhodiola coccinea* (Royle) Boriss.	*R*. *coccinea*
*Rhodiola atropurpurea* auct. non (Turcz.) Trautv. & C. A. Mey.	*R*. *atropurpurea*
*Rhodiola alsia* (Fröd.) S. H. Fu	*R*. *alsia*
*Rhodiola forrestii* (Raym.-Hamet) S. H. Fu	*R*. *forrestii*
*Rhodiola alterna* S. H. Fu	*R*. *alterna*
*Rhodiola atuntsuensis* (Praeger) S. H. Fu	*R*. *atuntsuensis*
*Rhodiola linearifolia* Boriss.	*R*. *linearifolia*
*Rhodiola purpureoviridis* subsp. *phariensis* (H. Ohba) H. Ohba	*R*. *purpureoviridis*
*Rhodiola pamiroalaica* Boriss.	*R*. *pamiroalaica*
*Rhodiola imbricata* Edgew.	*R*. *imbricata*
*Rhodiola litwinowii* Boriss.	*R*. *litwinowii*
*Rhodiola semenovii* (Regel & Herder) Boriss.	*R*. *semenovii*
*Rhodiola subopposita* (Maxim.) Jacobsen	*R*. *subopposita*
*Rhodiola primuloides* (Franch.) S. H. Fu	*R*. *primuloides*
*Rhodiola chrysanthemifolia* (H. Lév.) S. H. Fu	*R*. *chrysanthemifolia*
*Rhodiola sexifolia* S. H. Fu	*R*. *sexifolia*
*Rhodiola tieghemii* (Raym.-Hamet) S. H. Fu	*R*. *tieghemii*
*Rhodiola kashgarica* Boriss.	*R*. *kashgarica*
*Rhodiola junggarica* C. Y. Yang & N. R. Cui	*R*. *junggarica*
*Rhodiola serrata* H. Ohba	*R*. *serrata*
*Rhodiola recticaulis* Boriss.	*R*. *recticaulis*
*Rhodiola macrocarpa* (Praeger) S. H. Fu	*R*. *macrocarpa*
*Rhodiola sinuata* (Royle ex Edgew.) S. H. Fu	*R*. *sinuata*
*Rhodiola ishidae* (Miyabe & Kudô) Hara	*R*. *ishidae*

## 2 Materials and methods

A comprehensive literature search was conducted across multiple academic databases, including PubMed, CNKI, Web of Science, Elsevier, Baidu Scholar, and Springer, to retrieve studies relevant to the isolation, identification, and qualitative/quantitative analysis of chemical constituents in *Rhodiola* species. The search was concluded in May 2025. Only research articles published in English or Chinese were considered for inclusion. The reference lists of all retrieved articles were also manually screened to identify additional relevant publications.

Furthermore, to ensure the reliability of the compiled phytochemical data for *Rhodiola* species, compounds were excluded from this review if they were only tentatively identified via mass spectrometry and lacked unambiguous confirmation by reference standards.

All chemical structures presented in this review were drawn using ChemDraw 20.0.

## 3 Results

To date, phytochemical investigations have successfully isolated and identified over 500 compounds from the genus *Rhodiola.* Based on a comprehensive synthesis of compound classifications from numerous published studies on *Rhodiola* phytochemistry, these compounds are classified herein into eight distinct groups: phenylalkanoid derivatives, phenylpropanoids, flavonoids, terpenoids, phenolics and organic acids, cyanogenic glycosides, steroids, and other compounds (Tao et al., 2019; Li et al., 2017; Zhou et al., 2014; Ali et al., 2008; Olennikov and Chirikova, 2022). The vast majority of these compounds are accumulated in the roots and rhizomes, with a minor fraction isolated from aerial parts or callus tissues. In the following sections, key information about these compounds, including their chemical names, molecular formulas, chemical structures, and botanical sources, is systematically summarized.

### 3.1 Phenylalkanoid derivatives

**FIGURE 1:**
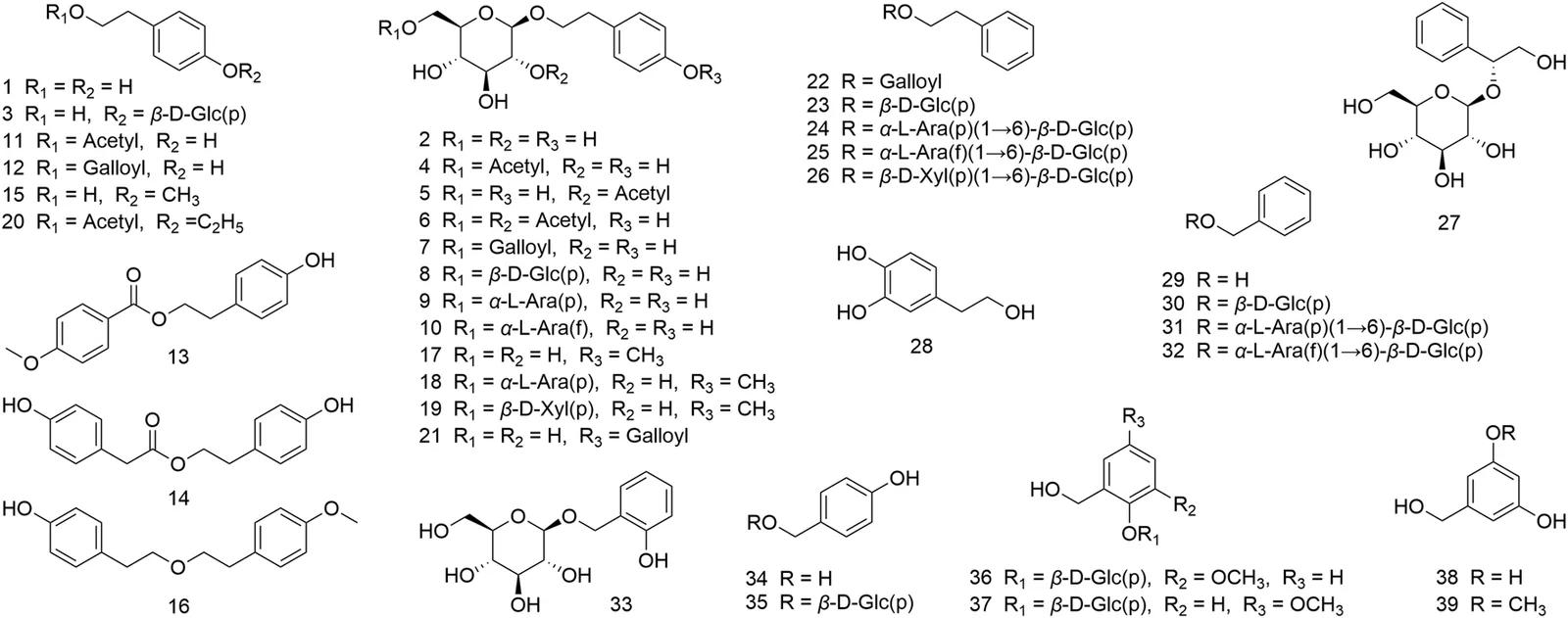
Structures of phenylalkanoid derivatives.

Phenylalkanoid derivatives, ubiquitously distributed in *Rhodiola* species, are the most emblematic class of secondary metabolites in the genus. A total of 39 compounds—primarily phenylethanol and benzyl alcohol derivatives—are summarized in this paper. Salidroside and its aglycone tyrosol are the most prominent, having been detected in over 30 chemically studied *Rhodiola* species. Salidroside, in particular, is recognized as a key bioactive constituent for the genus. The 2020 edition of the Chinese Pharmacopoeia specifies a minimum salidroside content of 0.5% in *R. crenulata* for medicinal use. The details of these compounds are presented in Table 2 and Figure 1.

**TABLE 2:** Phenylalkanoid derivatives from *Rhodiola* species.

No.	Compounds	Molecular formula	Source of species	References
1	Tyrosol	C_8_H_10_O_2_	*R. crenulata*, *R. rosea*, *R. kirilowii*, *R. fastigiata*, *R. wallichiana*, *R. tangutica*, *R. sachalinensis*, *R. yunnanensis*, *R. quadrifida*, *R. sacra*, *R. bupleuroides*, *R. henryi*, *R. himalensis*, *R. tibetica*, *R. heterodonta*, *R. gelida*, *R. coccinea*, *R. alsia*, *R. alterna*, *R. atuntsuensis*, *R. linearifolia*, *R. purpureoviridis*, *R. pamiroalaica*, *R. imbricata*, *R. litwinowii*, *R. chrysanthemifolia*, *R. sexifolia*, *R. tieghemii*, *R. kashgarica*, *R. junggarica*, *R. serrata*, *R. recticaulis*	Liu et al., 2013; Chen D. J. et al., 2012; Wang C. H. et al., 2020; Zuo et al., 2007; Yang et al., 2002; Ou et al., 2020; Nan et al., 2018; Choe et al., 2012; Chi et al., 2012; Altantsetseg et al., 2007; Qiu et al., 1991; Liu, 2003; Chen et al., 2013; Ma et al., 2008; Wang and Ruan, 2005; Olennikov et al., 2019; Shao et al., 2004; Chen et al., 1999; Ma et al., 1995; Choudhary et al., 2015a
2	Salidroside	C_14_H_20_O_7_	*R. crenulata*, *R. rosea*, *R. kirilowii*, *R. fastigiata*, *R. wallichiana*, *R. tangutica*, *R. sachalinensis*, *R. yunnanensis*, *R. dumulosa*, *R. quadrifida*, *R. sacra*, *R. bupleuroides*, *R. henryi*, *R. himalensis*, *R. tibetica*, *R. heterodonta*, *R. gelida*, *R. coccinea*, *R. atropurpurea*, *R. alsia*, *R. forrestii*, *R. alterna*, *R. atuntsuensis*, *R. linearifolia*, *R. purpureoviridis*, *R. pamiroalaica*, *R. imbricata*, *R. litwinowii*, *R. subopposita*, *R. primuloides*, *R. chrysanthemifolia*, *R. sexifolia*, *R. tieghemii*, *R. kashgarica*, *R. junggarica*, *R. serrata*, *R. recticaulis*, *R. macrocarpa*, *R. sinuata*	Liu et al., 2013; Chen D. J. et al., 2012; Wang C. H. et al., 2020; Nan et al., 2018; Choe et al., 2012; Chi et al., 2012; Ma et al., 2008; Wang and Ruan, 2005; Olennikov et al., 2019; Shao et al., 2004; Chen et al., 1999; Ma et al., 1995; Mao et al., 2007; Grace et al., 2009; Rattan et al., 2020; Zhu et al., 2024; Huang H. et al., 2008; Wiedenfeld et al., 2007a; An et al., 2007; Chen et al., 2003; Kang et al., 1998; Chen et al., 2008
3	Icariside D2	C_14_H_20_O_7_	*R. crenulata*, *R. rosea*, *R. sacra*, *R. recticaulis*	Chen D. J. et al., 2012; Olennikov et al., 2019; Lee et al., 2016; Daikonya and Kitanaka, 2011
4	6′-*O*-Acetylsalidroside	C_16_H_22_O_8_	*R. rosea*	Wang C. H. et al. (2020)
5	Rhodirosin L	C_16_H_22_O_8_	*R. rosea*	Olennikov and Chirikova (2024)
6	Rhodirosin M	C_18_H_24_O_9_	*R. rosea*	Olennikov and Chirikova (2024)
7	6′-*O*-Galloylsalidroside	C_21_H_24_O_11_	*R. crenulata*, *R. rosea*, *R. kirilowii*, *R. fastigiata*, *R. wallichiana*, *R. tangutica*, *R. sachalinensis*, *R. himalensis*	Wang C. H. et al., 2020; Zhu et al., 2024; Zhang et al., 2010; Fan et al., 2001; Song et al., 2018
8	2-(4-Hydroxyphenyl)ethyl-*O-β-*D-glucopyranosyl(1→6)*-β-*D-glucopyranoside	C_20_H_30_O_12_	*R. crenulata*	Chen D. J. et al. (2012)
9	Steviophethanoside	C_19_H_28_O_11_	*R. rosea*	Olennikov and Chirikova (2024)
10	Rhodirosin K	C_19_H_28_O_11_	*R. rosea*	Olennikov and Chirikova (2024)
11	2-(4-Hydroxyphenyl)ethyl acetate	C_10_H_12_O_3_	*R. rosea*	Wang C. H. et al. (2020)
12	2-(4-Hydroxyphenyl)ethyl 3,4,5-trihydroxybenzoate	C_15_H_14_O_6_	*R. crenulata*	Zhou et al. (2015)
13	*p*-Hydroxyphenethyl anisate	C_16_H_16_O_4_	*R. wallichiana*, *R. sachalinensis*	Zhao, 2016; Nakamura et al., 2007
14	Aspergillol B	C_16_H_16_O_4_	*R. rosea*, *R. wallichiana*	Lee et al., 2016; Zhao, 2016
15	4-Methoxyphenethyl alcohol	C_9_H_12_O_2_	*R. kirilowii*, *R. heterodonta*, *R. imbricata*	Choudhary et al., 2015a; Grace et al., 2009; Yang et al., 2011a
16	4-Hydroxyphenylethyl-(4′-methoxyphenylethyl)ether	C_17_H_20_O_3_	*R. kirilowii*	Yang et al. (2011a)
17	Viridoside	C_15_H_22_O_7_	*R. rosea*, *R. sachalinensis*, *R. heterodonta*	Grace et al., 2009; Nakamura et al., 2007; Ali et al., 2008
18	Mongrhoside	C_20_H_30_O_11_	*R. rosea*, *R. sachalinensis*, *R. quadrifida*, *R. heterodonta*, *R. gelida*, *R. recticaulis*	Olennikov et al., 2019; Grace et al., 2009; Wiedenfeld et al., 2007a; Ali et al., 2008; Avula et al., 2009
19	Heterodontoside	C_20_H_30_O_11_	*R. heterodonta*	Grace et al. (2009)
20	4-Ethoxyphenylethanol acetate	C_12_H_16_O_3_	*R. kirilowii*	Yang et al. (2011a)
21	Pharienside	C_21_H_24_O_11_	*R. purpureoviridis*	Ma et al. (1995)
22	Phenethyl gallate	C_15_H_14_O_5_	*R. sacra*	Daikonya and Kitanaka (2011)
23	2-Phenylethyl-1-*O*-*β*-D-glucopyranoside	C_14_H_20_O_6_	*R. crenulata*, *R. rosea*, *R. sachalinensis*, *R. sacra*	Nakamura et al., 2007; Nakamura et al., 2008; Ma, 2006; Fan et al., 1999
24	2-Phenylethyl-1-*O*-*α*-L-arabinopyranosyl(1→6)-*β*-D-glucopyranoside	C_19_H_28_O_10_	*R. crenulata*, *R. rosea*, *R. sachalinensis*, *R. sacra*, *R. recticaulis*	Olennikov et al., 2019; Ali et al., 2008; Avula et al., 2009; Nakamura et al., 2008; Fan et al., 1999
25	2-Phenylethyl-1-*O*-*α*-L-arabinofuranosyl(1→6)-*β*-D-glucopyranoside	C_19_H_28_O_10_	*R. crenulata*	Yang et al. (2013)
26	2-Phenylethyl-1-*O*-*β*-D-xylopyranosyl(1→6)-*β*-D-glucopyranoside	C_19_H_28_O_10_	*R. crenulata*	Yang et al. (2013)
27	Crenulatanoside C	C_14_H_20_O_7_	*R. crenulata*	Yang et al. (2012b)
28	Hydroxytyrosol	C_8_H_10_O_3_	*R. crenulata*	Ma et al. (2022)
29	Benzyl alcohol	C_7_H_8_O	*R. rosea*, *R. wallichiana*	Olennikov et al., 2020; Zhao et al., 2016
30	Benzyl-*O-β*-D-glucopyranoside	C_13_H_18_O_6_	*R. crenulata*, *R. rosea*, *R. wallichiana*, *R. sachalinensis*	Nakamura et al., 2007; Yang et al., 2013; Wang et al., 2015; Mudge et al., 2012
31	Benzyl-*O-α*-L-arabinopyranosyl(1→6)-*β*-D-glucopyranoside	C_18_H_26_O_10_	*R. rosea*, *R. sachalinensis*	Nakamura et al., 2007; Ali et al., 2008
32	Benzyl-*O-α*-L-arabinofuranosyl(1→6)-*β*-D-glucopyranoside	C_18_H_26_O_10_	*R. rosea*, *R. sachalinensis*	Nakamura et al., 2007; Ali et al., 2008
33	Salicin	C_13_H_18_O_7_	*R. rosea*	Liu et al. (2024b)
34	*p*-Hydroxybenzyl alcohol	C_7_H_8_O_2_	*R. crenulata*, *R. imbricata*	Choudhary et al., 2015a; Yan et al., 2021
35	4-Hydroxybenzyl *β*-D-glucopyranoside	C_13_H_18_O_7_	*R. crenulata*	Nakamura et al. (2008)
36	2-Hydroxymethyl-6-methoxyphenyl-*β*-D-glucopyranoside	C_14_H_20_O_8_	*R. imbricata*	Choudhary et al. (2015a)
37	2-Hydroxymethyl-4-methoxyphenyl-*β-D*-glucopyranoside	C_14_H_20_O_8_	*R. imbricata*	Choudhary et al., 2015a; Choudhary et al., 2015b
38	3,5-Dihydroxybenzyl alcohol	C_7_H_8_O_3_	*R. imbricata*	Choudhary et al. (2015a)
39	3-Hydroxy-5-methoxybenzyl alcohol	C_8_H_10_O_3_	*R. imbricata*	Choudhary et al., 2015a; Choudhary et al., 2015b

### 3.2 Phenylpropanoids

**FIGURE 2:**
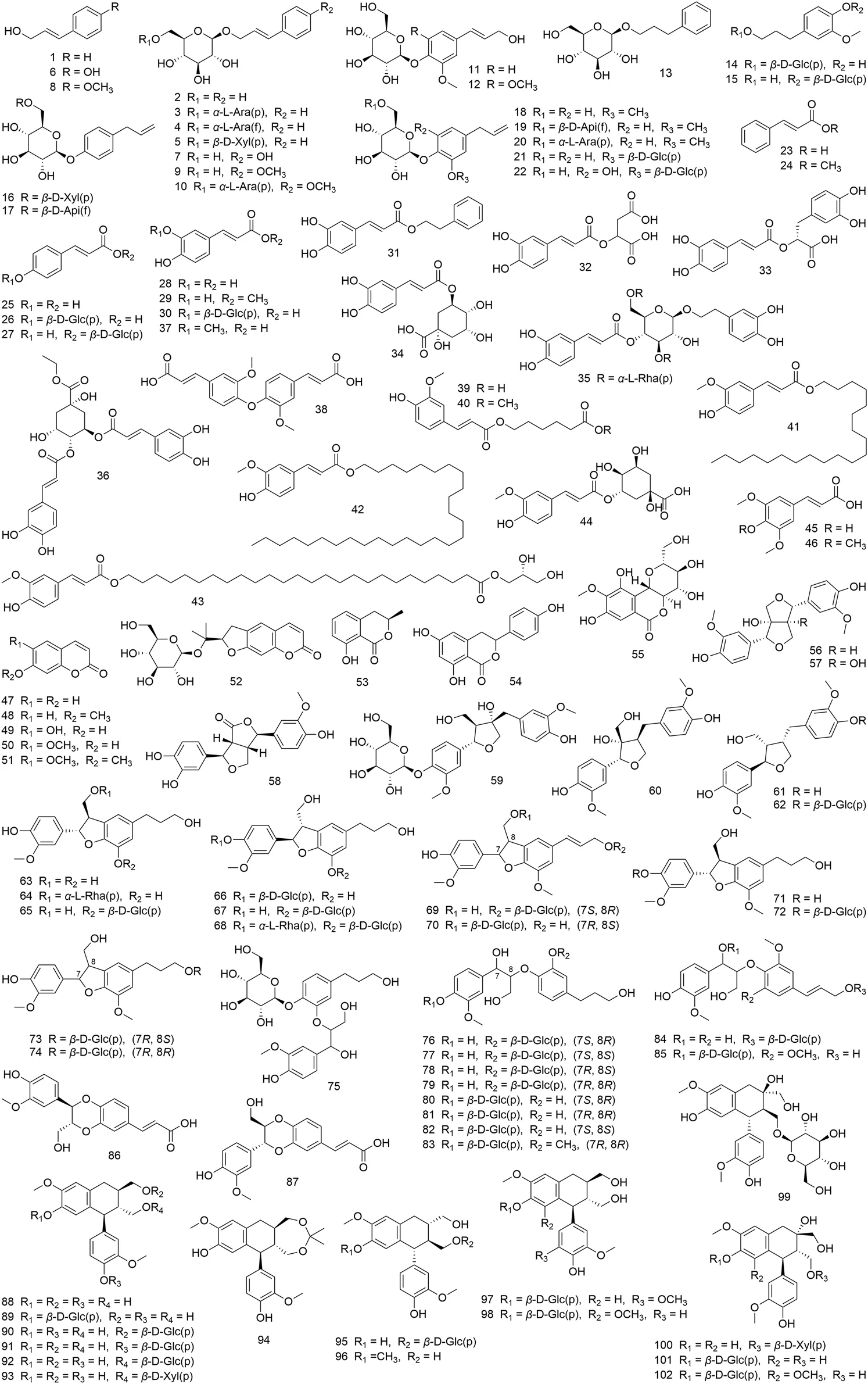
Structures of phenylpropanoids.

Phenylpropanoids represent another important class of bioactive constituents in *Rhodiola* species, structurally subdivided into three groups: simple phenylpropanoids (1–46), coumarins (47–55), and lignans (56–102). Among the simple phenylpropanoids, cinnamyl alcohol derivatives—particularly rosavin, rosarin, and rosin (collectively termed “rosavins”)—are regarded as chemical diagnostic markers for *R. rosea*. Coumarins are the fewest in number, with only nine identified compounds reported in *Rhodiola* species to date. Major lignans have core structures based on pinoresinol, olivil, lariciresinol, dehydrodiconiferyl alcohol, isolariciresinol, and cycloolivil. Most of the 47 lignan compounds identified in the genus were isolated from *R. crenulata*. The details of these compounds are presented in Table 3 and Figure 2.

**TABLE 3:** Phenylpropanoids from *Rhodiola* species.

No.	Compounds	Molecular formula	Source of species	References
1	Cinnamyl alcohol	C_9_H_10_O	*R. rosea*, *R. sachalinensis*, *R. quadrifida*	Altantsetseg et al., 2007; van Diermen et al., 2009; Song et al., 2003
2	Rosin	C_15_H_20_O_6_	*R. crenulata*, *R. rosea*, *R. tangutica*, *R. sachalinensis*, *R. quadrifida*, *R. himalensis*, *R. tibetica*	Liu et al., 2013; Nan et al., 2018; Altantsetseg et al., 2007; Ma et al., 2008; Mudge et al., 2012; Liu et al., 2024a
3	Rosavin	C_20_H_28_O_10_	*R. crenulata*, *R. rosea*, *R. kirilowii*, *R. sachalinensis*, *R. yunnanensis*, *R. quadrifida*, *R. himalensis*, *R. tibetica*, *R. alterna*, *R. imbricata*, *R. serrata*	Liu et al., 2013; Ma et al., 2008; Shao et al., 2004; Rattan et al., 2020; Zhu et al., 2024; Wiedenfeld et al., 2007a; Mudge et al., 2012; Han et al., 2016
4	Rosarin	C_20_H_28_O_10_	*R. crenulata*, *R. rosea*, *R. kirilowii*, *R. sachalinensis*, *R. quadrifida*, *R. himalensis*, *R. tibetica*, *R. imbricata*	Liu et al., 2013; Altantsetseg et al., 2007; Ma et al., 2008; Rattan et al., 2020; Mudge et al., 2012; Cui et al., 2016
5	Cinnamyl-(6′-*O-β*-D-xylopyranosyl)-*O-β-D*-glucopyranoside	C_20_H_28_O_10_	*R. rosea*, *R. sachalinensis*	Tolonen et al., 2003; Ni, 2016
6	*p*-Coumaryl alcohol	C_9_H_10_O_2_	*R. rosea*	Kurkin et al. (1991)
7	Triandrin	C_15_H_20_O_7_	*R. crenulata*, *R. rosea*	Nakamura et al., 2008; Tolonen et al., 2003
8	*p*-Methoxycinnamyl alcohol	C_10_H_12_O_2_	*R. rosea*	Kurkin et al. (1991)
9	Vimalin	C_16_H_22_O_7_	*R. crenulata*, *R. rosea*	Nakamura et al., 2008; Tolonen et al., 2003
10	4-Methoxy-cinnamyl-(6′-*O-α*-L-arabinopyranosyl)-*O-β*-D-glucopyranoside	C_21_H_30_O_11_	*R. rosea*	Tolonen et al. (2003)
11	Coniferin	C_16_H_22_O_8_	*R. crenulata*	Nakamura et al. (2008)
12	Syringin	C_17_H_24_O_9_	*R. crenulata*	Liu et al. (2024a)
13	3-Phenylpropyl *β*-D-glucopyranoside	C_15_H_22_O_6_	*R. sachalinensis*	Nakamura et al. (2007)
14	Dihydroconiferyl alcohol γ-*O-β*-D-glucopyranoside	C_16_H_24_O_8_	*R. fastigiata*	Yang et al. (2002)
15	Dihydroconiferin	C_16_H_24_O_8_	*R. crenulata*, *R. wallichiana*	Nakamura et al., 2008; Wang et al., 2015
16	Miyaginin	C_20_H_28_O_10_	*R. wallichiana*	Chai et al. (2015)
17	Chavicol 4-*O-β*-D-apiofuranosyl(1→6)-*β*-D-glucopyranoside	C_20_H_28_O_10_	*R. wallichiana*	Chai et al. (2015)
18	Citrusin C	C_16_H_22_O_7_	*R. crenulata*	Liu et al. (2024a)
19	Eugenyl-*O-β*-D-apiofuranosyl(1→6)-*β*-D-glucopyranoside	C_21_H_30_O_11_	*R. wallichiana*, *R. tangutica*, *R. sachalinensis*	Zhang et al., 2010; Nakamura et al., 2007; Chai et al., 2015
20	Gein	C_21_H_30_O_11_	*R. tangutica*, *R. sachalinensis*	Zhang et al., 2010; Nakamura et al., 2007
21	4-Allylcatechol 1,2-di-*O-β*-D-glucopyranoside	C_21_H_30_O_12_	*R. crenulata*	Liu et al. (2024a)
22	5-Allylpyrogallol 1,2-di-*O-β*-D-glucopyranoside	C_21_H_30_O_13_	*R. crenulata*	Liu et al. (2024a)
23	Cinnamic acid	C_9_H_8_O_2_	*R. rosea*, *R. imbricata*	Rattan et al., 2020; Olennikov et al., 2020
24	Methyl cinnamate	C_10_H_10_O_2_	*R. wallichiana*	Zhao (2016)
25	*p*-Coumaric acid	C_9_H_8_O_3_	*R. crenulata*, *R. rosea*, *R. kirilowii*, *R. fastigiata*, *R. wallichiana*, *R. tangutica*, *R. sachalinensis*, *R. sacra*, *R. bupleuroides*, *R. imbricata*	Nan et al., 2018; Rattan et al., 2020; Lee et al., 2016; Daikonya and Kitanaka, 2011; Nakamura et al., 2007; Liu et al., 2019; Chen et al., 2021; Li et al., 2021; Dong et al., 2009; Huang Y. J. et al., 2008
26	*p*-Coumaric acid 4-*O-β*-D-glucopyranoside	C_15_H_18_O_8_	*R. crenulata*, *R. rosea*, *R. wallichiana*	Ou et al., 2020; Yang et al., 2013; Ma et al., 2012
27	1-*O-p*-Coumaroylglucose	C_15_H_18_O_8_	*R. rosea*	Kurkin et al. (1991)
28	Caffeic acid	C_9_H_8_O_4_	*R. crenulata*, *R. rosea*, *R. kirilowii*, *R. fastigiata*, *R. wallichiana*, *R. tangutica*, *R. sachalinensis*, *R. sacra*, *R. himalensis*, *R. purpureoviridis*	Ou et al., 2020; Nan et al., 2018; Ma et al., 1995; Zhu et al., 2024; Lee et al., 2016; Nakamura et al., 2007; Nakamura et al., 2008; Fan et al., 1999; Li et al., 2021
29	Methyl caffeate	C_10_H_10_O_4_	*R. crenulata*	Chen et al. (2024)
30	Caffeic acid 3′-*O*-*β*-D-glucopyranoside	C_15_H_18_O_9_	*R. rosea*	Kurkin et al. (1991)
31	Caffeic acid phenethyl ester	C_17_H_16_O_4_	*R. sacra*	Jung et al. (2008)
32	(*E*)-Caffeoylmalic acid	C_13_H_12_O_8_	*R. rosea*	Iwashina et al. (2024)
33	Rosmarinic acid	C_18_H_16_O_8_	*R. rosea*	Wang F. Y. et al. (2007)
34	Chlorogenic acid	C_16_H_18_O_9_	*R. rosea*, *R. kirilowii*, *R. quadrifida*	Wiedenfeld et al., 2007a; Gryszczyńska et al., 2013
35	Poliumoside	C_35_H_46_O_19_	*R. crenulata*	Liu et al. (2024a)
36	Ethyl 4,5-dicaffeoylquinate	C_27_H_28_O_12_	*R. crenulata*	Qu et al. (2020)
37	Ferulic acid	C_10_H_10_O_4_	*R. crenulata*, *R. rosea*, *R. fastigiata*, *R. wallichiana*, *R. tangutica*, *R. heterodonta*	Nan et al., 2018; Zhao et al., 2016; Li et al., 2021; Chen et al., 2024; Krasnov et al., 1976; Polumackanycz et al., 2022
38	3-Methoxy-4-feruloyloxy cinnamic acid	C_20_H_18_O_7_	*R. crenulata*	Liu et al. (2024a)
39	6-Feruloyloxyhexanoic acid	C_16_H_20_O_6_	*R. wallichiana*	Song et al. (2018)
40	Rhodiolate	C_17_H_22_O_6_	*R. crenulata*	Zhou et al. (2015)
41	Eicosyl ferulate	C_30_H_50_O_4_	*R. rosea*	Michels et al. (2020)
42	Octacosyl (*E*)-ferulate	C_38_H_66_O_4_	*R. crenulata*, *R. yunnanensis*, *R. henryi*	Li et al., 2012; Guo et al., 1993; Lou and Zuo, 1991
43	2(*R*)-26-{[(2*E*)-3-(4-Hydroxy-3-methoxyphenyl)-1-oxo-2-propen-1-yl]oxy}-2,3-dihydroxypropyl ester	C_39_H_66_O_8_	*R. sachalinensis*	Ding et al. (2018)
44	3-*O*-Feruloylquinic acid	C_17_H_20_O_9_	*R. rosea*	Olennikov et al. (2020)
45	Sinapic acid	C_11_H_12_O_5_	*R. rosea*	Polumackanycz et al. (2022)
46	3,4,5-Trimethoxycinnamic acid	C_12_H_14_O_5_	*R. crenulata*, *R. kirilowii*, *R. fastigiata*, *R. himalensis*	Zhu et al., 2024; Chen et al., 2021
47	Umbelliferone	C_9_H_6_O_3_	*R. quadrifida*, *R. sacra*, *R. heterodonta*, *R. coccinea*, *R. purpureoviridis*	Qiu et al., 1991; Ma et al., 1995; Krasnov et al., 1976; Krasnov and Khoruzhaya, 1974
48	7-Methoxycoumarin	C_10_H_8_O_3_	*R. rosea*	Ma et al. (2012)
49	Esculetin	C_9_H_6_O_4_	*R. linearifolia*	Kurlin and Zapesochnaya (1986)
50	Scopoletin	C_10_H_8_O_4_	*R. quadrifida*, *R. coccinea*	Krasnov and Khoruzhaya (1974)
51	Scoparone	C_11_H_10_O_4_	*R. crenulata*	Liu et al. (2024a)
52	Nodakenin	C_20_H_24_O_9_	*R. sachalinensis*	Nakamura et al. (2007)
53	(*R*)-Mellein	C_10_H_10_O_3_	*R. kirilowii*, *R. wallichiana*	Zhao, 2016; Yang et al., 2011a
54	Thunberginol C	C_15_H_12_O_5_	*R. sacra*	Daikonya and Kitanaka (2011)
55	Bengenin	C_14_H_16_O_9_	*R. wallichiana*	Ou et al. (2020)
56	8-Hydroxypinoresinol	C_20_H_22_O_7_	*R. wallichiana*	Zhao (2016)
57	Prinsepiol	C_20_H_22_O_8_	*R. crenulata*	Qu et al. (2020)
58	(+)-Syzygiresinol A	C_19_H_18_O_7_	*R. crenulata*	Zhou et al. (2015)
59	Olivil 4-*O*-*β*-D-glucopyranoside	C_26_H_34_O_12_	*R. crenulata*	Yang et al. (2012a)
60	(−)-Berchemol	C_20_H_24_O_7_	*R. wallichiana*	Chai et al. (2015)
61	(−)-Lariciresinol	C_20_H_24_O_6_	*R. rosea*	Kurkin et al. (1991)
62	(−)-Lariciresinol 4′-*O-β*-D-glucopyranoside	C_26_H_34_O_11_	*R. rosea*	Kurkin et al. (1991)
63	Cedrusin	C_19_H_22_O_6_	*R. fastigiata*	Yang et al. (2002)
64	(7*S*,8*R*)-3′,4,9,9′-Tetrahydroxy-3-methoxy-4′,7-epoxy-5′,8-lignan 9-*O*-*α*-L-rhamnopyranoside	C_25_H_32_O_10_	*R. crenulata*	Yang et al. (2012a)
65	Clemastanin A	C_25_H_32_O_11_	*R. crenulata*	Yang et al. (2012a)
66	(7*R*,8*S*)-3′,4,9,9′-Tetrahydroxy-3-methoxy-4′,7-epoxy-5′,8-lignan 4-*O*-*β*-D-glucopyranoside	C_25_H_32_O_11_	*R. crenulata*	Yang et al. (2012a)
67	(7*R*,8*S*)-3′,4,9,9′-Tetrahydroxy-3-methoxy-4′,7-epoxy-5′,8-lignan 3′-*O-β*-D-glucopyranoside	C_25_H_32_O_11_	*R. crenulata*	Yang et al. (2012a)
68	(7*R*,8*S*)-3′,4,9,9′-Tetrahydroxy-3-methoxy-4′,7-epoxy-5′,8-lignan 3′-*O-β*-D-glucopyranoside-4-*O-α*-L-rhamnopyranoside	C_31_H_42_O_15_	*R. crenulata*	Yang et al. (2012a)
69	(7*S*,8*R*)-Dehydrodiconiferyl alcohol 9′-*O*-*β*-D-glucopyranoside	C_26_H_32_O_11_	*R. crenulata*	Liu et al. (2024a)
70	(7*R*,8*S*)-Dehydrodiconiferyl alcohol 9-*O*-*β*-D-glucopyranoside	C_26_H_32_O_11_	*R. crenulata*	Liu et al. (2024a)
71	(+)-Dihydrodehydrodiconiferyl alcohol	C_20_H_24_O_6_	*R. crenulata*	Zhou et al. (2015)
72	(7*S*,8*R*)-Dihydrodehydrodiconiferyl alcohol 4-*O*-*β*-D-glucopyranoside	C_26_H_34_O_11_	*R. crenulata*	Liu et al. (2024a)
73	(7*R*,8*S*)-Dihydrodehydrodiconiferyl alcohol 9′-*O*-*β*-D-glucopyranoside	C_26_H_34_O_11_	*R. crenulata*	Liu et al. (2024a)
74	(7*R*,8*R*)-Dihydrodehydrodiconiferyl alcohol 9′-*O*-*β*-D-glucopyranoside	C_26_H_34_O_11_	*R. crenulata*	Liu et al. (2024a)
75	4′,7′,9,9′-Tetrahydroxy-3′-methoxy-3,8′-oxyneolignan 4-*O-β*-D-glucopyranoside	C_25_H_34_O_12_	*R. rosea*	Ma (2006)
76	(7*S*,8*R*)-4,7,9,3′,9′-Pentahydroxy-3-methoxyl-8-4′-oxyneolignan 3′-*O-β*-D-glucopyranoside	C_25_H_34_O_12_	*R. crenulata*	Yang et al. (2012a)
77	(7*S*,8*S*)-4,7,9,3′,9′-Pentahydroxy-3-methoxy-8-4′-oxyneolignan 3′-*O-β*-D-glucopyranoside	C_25_H_34_O_12_	*R. crenulata*	Yang et al. (2012a)
78	(7*R*,8*S*)-4,7,9,3′,9′-Pentahydroxy-3-methoxy-8-4′-oxyneolignan 3′-*O-β-D*-glucopyranoside	C_25_H_34_O_12_	*R. crenulata*	Yang et al. (2012a)
79	(7*R*,8*R*)-4,7,9,3′,9′-Pentahydroxy-3-methoxy-8-4′-oxyneolignan 3′-*O-β*-D-glucopyranoside	C_25_H_34_O_12_	*R. crenulata*	Yang et al. (2012a)
80	(7*S*,8*R*)-4,7,9,3′,9′-Pentahydroxy-3-methoxy-8-4′-oxyneolignan 4-*O-β-D*-glucopyranoside	C_25_H_34_O_12_	*R. crenulata*	Yang et al. (2012a)
81	(7*R*,8*R*)-4,7,9,3′,9′-Pentahydroxy-3-methoxy-8-4′-oxyneolignan 4-*O-β*-D-glucopyranoside	C_25_H_34_O_12_	*R. crenulata*	Yang et al. (2012a)
82	(7*S*,8*S*)-4,7,9,3′,9′-Pentahydroxy-3-methoxy-8-4′-oxyneolignan 4-*O-β*-D-glucopyranoside	C_25_H_34_O_12_	*R. crenulata*	Yang et al. (2012a)
83	(7*R*,8*R*)-Threo-4,7,9,9′-tetrahydroxy-3,3′-dimethoxy-8-O-4′-neolignan 4-*O-β-D*-glucopyranoside	C_26_H_36_O_12_	*R. crenulata*	Yang et al. (2012a)
84	Hyuganoside IIIa	C_26_H_34_O_12_	*R. crenulata*	Liu et al. (2024a)
85	3-Hydroxy-1-(4-hydroxy-3-methoxyphenyl)-2-[4-(3-hydroxy-1-(*E*)-propenyl)-2,6-dimethoxyphenoxy]propyl-*β*-D-glucopyranoside	C_27_H_36_O_13_	*R. crenulata*	Liu et al. (2024a)
86	(7*R*,8*R*)-3-Methoxy-8′-carboxy-7′-en-3′,8-epoxy-7,4′-oxyneolignan-4,9-diol	C_19_H_18_O_7_	*R. crenulata*	Zhou et al. (2015)
87	(7*R*,8*R*)-3-Methoxy-8′-carboxy-7′-en-3′,7-epoxy-8,4′-oxyneolignan-4,9-diol	C_19_H_18_O_7_	*R. crenulata*	Zhou et al. (2015)
88	(+)-Isolariciresinol	C_20_H_24_O_6_	*R. crenulata*, *R. fastigiata*	Yang et al., 2002; Yang et al., 2012a
89	(+)-Isolariciresinol 4-*O-β*-D-glucopyranoside	C_26_H_34_O_11_	*R. crenulata*	Yang et al. (2012a)
90	(+)-Isolariciresinol 9-*O-β*-D-glucopyranoside	C_26_H_34_O_11_	*R. kirilowii*, *R. fastigiata*	Yang et al., 2002; Huang Y. J. et al., 2008
91	(+)-Isolariciresinol 4′-*O-β*-D-glucopyranoside	C_26_H_34_O_11_	*R. crenulata*	Yang et al. (2012a)
92	(+)-Isolariciresinol 9′-*O-β*-D-glucopyranoside	C_26_H_34_O_11_	*R. dumulosa*	Liu et al. (2008)
93	Schizandriside	C_25_H_32_O_10_	*R. crenulata*, *R. tangutica*, *R. yunnanensis*, *R. bupleuroides*	Zhang et al., 2010; Dong et al., 2009; Guo et al., 1993; Yang et al., 2012a
94	(+)-Isolariciresinol-9,9′-acetonide	C_23_H_28_O_6_	*R. crenulata*	Yang et al. (2013)
95	(−)-Isolariciresinol 9′-*O-β*-D-glucopyranoside	C_26_H_34_O_11_	*R. dumulosa*	Liu et al. (2008)
96	Scaphopetalone	C_21_H_26_O_6_	*R. wallichiana*	Chai et al. (2015)
97	5′-Methoxy-(+)-isolariciresinol 4-*O-β*-D-glucopyranoside	C_27_H_36_O_12_	*R. crenulata*	Yang et al. (2012a)
98	5-Methoxy-(+)-isolariciresinol 4-*O-β-D*-glucopyranoside	C_27_H_36_O_12_	*R. crenulata*	Yang et al. (2012a)
99	(−)-Cycloolivil 9′-*O-β*-D-glucopyranoside	C_26_H_34_O_12_	*R. sacra*	Daikonya and Kitanaka (2011)
100	(+)-Cycloolivil 9′-*O-β*-D-xylopyranoside	C_25_H_32_O_11_	*R. crenulata*	Yang et al. (2012a)
101	(+)-Cycloolivil 4-*O-β*-D-glucopyranoside	C_26_H_34_O_12_	*R. crenulata*	Yang et al. (2012a)
102	5-Methoxy-(+)-cycloolivil 4-*O-β*-D-glucopyranoside	C_27_H_36_O_13_	*R. crenulata*	Yang et al. (2012a)

### 3.3 Flavonoids

**FIGURE 3:**
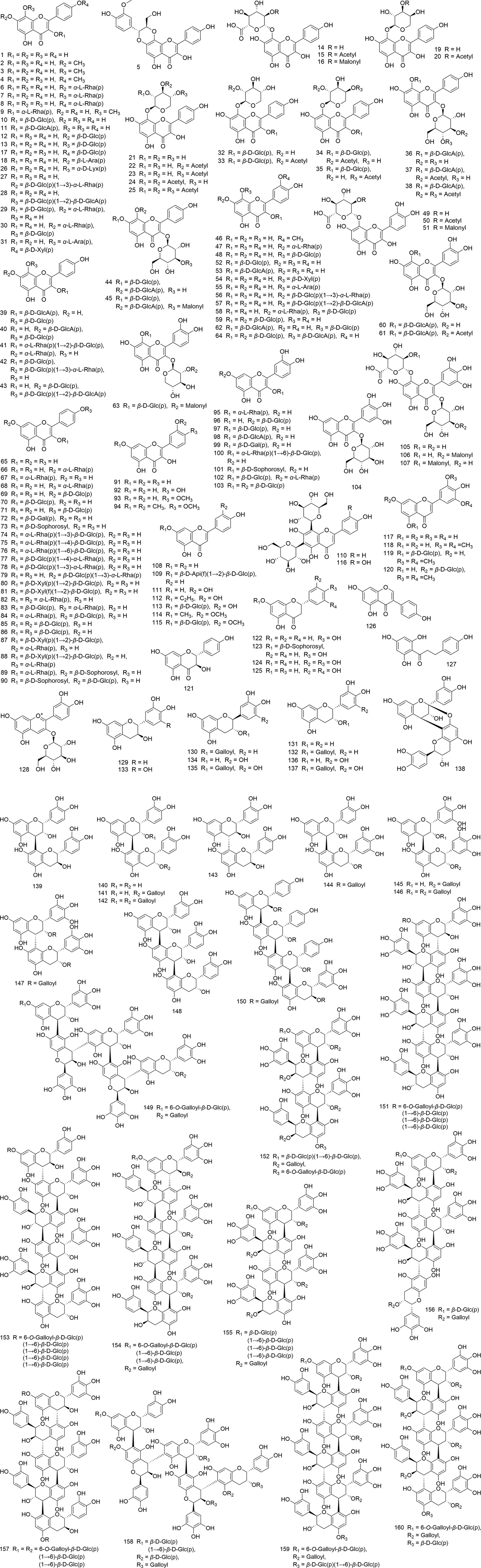
Structures of flavonoids.

Our investigations reveal that flavonoids constitute the largest and most structurally diverse class of phytochemicals in *Rhodiola* species, with 160 compounds isolated and identified. The details of these compounds are presented in Table 4 and Figure 3.

**TABLE 4:** Flavonoids from *Rhodiola* species.

No.	Compounds	Molecular formula	Source of species	References
1	Herbacetin	C_15_H_10_O_7_	*R. crenulata*, *R. rosea*, *R. wallichiana*, *R. sachalinensis*, *R. litwinowii*	Song et al., 2018; Chen et al., 2024; Zhang et al., 2016; Ma et al., 2014; Krasnov, 1979
2	Herbacetin 7-methyl ether	C_16_H_12_O_7_	*R. crenulata*	Zhou et al. (2015)
3	Herbacetin 8-methyl ether	C_16_H_12_O_7_	*R. rosea*, *R. dumulosa*, *R. bupleuroides*, *R. atuntsuensis*	Chen et al., 1999; Kurkin et al., 1984; Luo et al., 2005; Wang H. et al., 2016
4	Herbacetin 4′-methyl ether	C_16_H_12_O_7_	*R. fastigiata*, *R. bupleuroides*	Yang et al., 2002; Wang H. et al., 2016
5	Rhodiolin	C_25_H_20_O_10_	*R. crenulata*, *R. rosea*, *R. fastigiata*, *R. wallichiana*, *R. sachalinensis*, *R. quadrifida*, *R. bupleuroides*	Wiedenfeld et al., 2007a; Wang H. et al., 2016; Peng et al., 1996; Lee et al., 2000; Wang Y. S. et al., 2020; Zhang, 2021
6	Rhodionin	C_21_H_20_O_11_	*R. crenulata*, *R. rosea*, *R. kirilowii*, *R. fastigiata*, *R. wallichiana*, *R. sachalinensis*, *R. yunnanensis*, *R. dumulosa*, *R. sacra*, *R. henryi*, *R. alsia*, *R. forrestii*, *R. chrysanthemifolia*, *R. sexifolia*, *R. serrata*, *R*. *macrocarpa*	Liu et al., 2013; Choe et al., 2012; Daikonya and Kitanaka, 2011; Song et al., 2018; Zhou et al., 2015; Ma et al., 2014; Wang H. L. et al., 2006; Li and Zhang, 2008; Wang J. X. et al., 2006
7	Litvinolin	C_21_H_20_O_11_	*R. bupleuroides*, *R. litwinowii*, *R. ishidae*	Krasnov, 1979; Wang H. et al., 2016; Iwashina et al., 2023
8	Rhodiolatuntoside	C_21_H_20_O_11_	*R. crenulata*, *R. atuntsuensis*	Chen et al., 1999; Ma and Guo, 2007
9	Kaempferol-8-methoxy-3-*O-α*-L-rhamnopyranoside	C_22_H_22_O_11_	*R. rosea*	Wang C. H. et al. (2020)
10	Herbacetin 3-*O-β*-D-glucopyranoside	C_21_H_20_O_12_	*R. crenulata*, *R. rosea*, *R. sachalinensis*	Ni, 2016; Iwashina et al., 2024; Ni et al., 2016
11	Herbacetin 3-*O-β*-D-glucuronopyranoside	C_21_H_18_O_13_	*R. tangutica*	Olennikov and Prokopyev (2024)
12	Herbacetin 7-*O-β*-D-glucopyranoside	C_21_H_20_O_12_	*R. crenulata*, *R. wallichiana*, *R. sachalinensis*	Ni, 2016; Ni et al., 2016; Wang X. J. et al., 2016
13	Herbacin	C_21_H_20_O_12_	*R. rosea*	Ma et al. (2013)
14	Melocorin/Alginin	C_21_H_18_O_13_	*R. rosea*, *R. tangutica*, *R. quadrifida*, *R. ishidae*	Iwashina et al., 2024; Iwashina et al., 2023; Zapesochnaya, 1978; Olennikov and Chirikova, 2020
15	Rhodirosin F	C_23_H_20_O_14_	*R. rosea*	Olennikov and Chirikova (2022)
16	Rhodirosin E	C_24_H_20_O_16_	*R. rosea*	Olennikov and Chirikova (2022)
17	Gelidolin	C_21_H_20_O_12_	*R. gelida*	Krasnov and Alekseyuk (1979)
18	Gelolin	C_20_H_18_O_11_	*R. gelida*	Krasnov and Alekseyuk (1979)
19	Rhodalgin	C_20_H_18_O_11_	*R. fastigiata*, *R. tangutica*, *R. quadrifida*	Olennikov and Chirikova, 2020; Chen et al., 1991; Pangarova and Zapesochnaya, 1976
20	Acetylrhodalgin	C_22_H_20_O_12_	*R. rosea*, *R. tangutica*	Kurkin et al., 1984; Pangarova and Zapesochnaya, 1976
21	Rhodalin	C_20_H_18_O_11_	*R. rosea*, *R. quadrifida*, *R. tangutica*	Olennikov et al., 2020; Zapesochnaya, 1978; Olennikov and Chirikova, 2020
22	Acetylrhodalin	C_22_H_20_O_12_	*R. tangutica*	Zapesochnaya (1978)
23	Rhodiolgin D	C_22_H_20_O_12_	*R. tangutica*	Olennikov and Prokopyev (2024)
24	Diacetylrhodalgin	C_24_H_22_O_13_	*R. tangutica*	Pangarova and Zapesochnaya (1976)
25	Triacetylrhodalgin	C_26_H_24_O_14_	*R. tangutica*	Pangarova and Zapesochnaya (1976)
26	Herbacetin 8-*O-α*-D-lyxopyranoside	C_20_H_18_O_11_	*R. fastigiata*	Peng et al. (1996)
27	Rhodiosin	C_27_H_30_O_16_	*R. crenulata*, *R. rosea*, *R. wallichiana*, *R. tangutica*, *R. sachalinensis*, *R. yunnanensis*, *R. dumulosa*, *R. quadrifida*, *R. sacra*, *R. bupleuroides*	Nan et al., 2018; Zhu et al., 2024; Song et al., 2018; Zhou et al., 2015; Nakamura et al., 2007; Ma et al., 2014; Wang H. et al., 2016; Wang J. X. et al., 2006; Olennikov and Chirikova, 2020
28	Rhodiquadrin C	C_27_H_28_O_18_	*R. rosea*, *R. tangutica*, *R. quadrifida*	Olennikov and Prokopyev, 2024; Olennikov and Chirikova, 2020; Olennikov, 2023
29	Herbacetin 3-*O-β*-D-glucopyranoside-7-*O-α*-L-rhamnopyranoside	C_27_H_30_O_16_	*R. rosea*, *R. sachalinensis*, *R. ishidae*	Iwashina et al., 2023; Ma et al., 2013; Ni et al., 2013
30	Rhodionidin	C_27_H_30_O_16_	*R. rosea*	Olennikov (2023)
31	Rhodalide	C_25_H_26_O_15_	*R. tangutica*	Krasnov and Demidenko (1979)
32	Rhodalidin	C_26_H_28_O_16_	*R. rosea*, *R. tangutica*	Olennikov and Prokopyev, 2024; Zapesochnaya et al., 1985
33	Herbacetin 3-*O-β*-D-glucopyranoside-8-*O-*(2″-*O*-acetyl)-*β*-D-xylopyranoside	C_28_H_30_O_17_	*R. tangutica*	Olennikov and Prokopyev (2024)
34	Rhodiolgin B	C_28_H_30_O_17_	*R. tangutica*	Olennikov and Prokopyev (2024)
35	Rhodiolgin C	C_28_H_30_O_17_	*R. tangutica*	Olennikov and Prokopyev (2024)
36	Herbacetin 3-*O*-*β*-D-glucopyranoside-8-*O-β*-D-glucuronopyranoside	C_27_H_28_O_18_	*R. rosea*, *R. tangutica*, *R. quadrifida*	Iwashina et al., 2024; Olennikov and Prokopyev, 2024; Olennikov and Chirikova, 2020
37	Herbacetin 3-*O*-(3″-*O*-acetyl)-*β*-D-glucopyranoside-8-*O-β*-D-glucuronopyranoside	C_29_H_30_O_19_	*R. rosea*, *R. quadrifida*	Olennikov and Chirikova, 2020; Olennikov, 2023
38	Rhodirosin B	C_31_H_32_O_20_	*R. rosea*	Olennikov and Chirikova (2022)
39	Herbacetin 3-*O-β*-D-glucuronopyranoside-8-*O-β*-D-glucopyranoside	C_27_H_28_O_18_	*R. ishidae*	Iwashina et al. (2023)
40	Herbacetin 7-*O-β*-D-glucuronopyranoside-8-*O-β*-D-glucopyranoside	C_27_H_28_O_18_	*R. ishidae*	Iwashina et al. (2023)
41	Herbacetin 3-*O*-[*α*-L-rhamnopyranosyl(1→2)-*β*-D-glucopyranoside]-7-*O-α*-L-rhamnopyranoside	C_33_H_40_O_20_	*R. sachalinensis*	Han et al. (2011)
42	Sachaloside IV	C_33_H_40_O_21_	*R. sachalinensis*	Nakamura et al. (2007)
43	Rhodiolgin A	C_33_H_38_O_23_	*R. tangutica*	Olennikov and Prokopyev (2024)
44	Herbacetin 3,7-di-*O-β*-D-glucopyranoside-8-*O-β*-D-glucuronopyranoside	C_33_H_38_O_23_	*R. rosea*, *R. ishidae*	Iwashina et al., 2024; Iwashina et al., 2023
45	Rhodirosin A	C_36_H_40_O_26_	*R. rosea*	Olennikov and Chirikova (2022)
46	3,5,7,8,4′-Pentahydroxy-3′-methoxyflavone	C_16_H_12_O_8_	*R. bupleuroides*	Wang H. et al. (2016)
47	Rhodiolgin	C_21_H_20_O_12_	*R. rosea*, *R. kirilowii*, *R. quadrifida*	Huang Y. J. et al., 2008; Yoshikawa et al., 1996; Ming et al., 2005
48	Gossypin	C_21_H_20_O_13_	*R. rosea*, *R. purpureoviridis*	Ma et al., 1995; Olennikov et al., 2020
49	Hibifolin	C_21_H_18_O_14_	*R. rosea*, *R. tangutica*, *R. quadrifida*, *R. ishidae*	Iwashina et al., 2024; Iwashina et al., 2023; Olennikov and Prokopyev, 2024; Olennikov and Chirikova, 2020
50	Rhodirosin D	C_23_H_20_O_15_	*R. rosea*	Olennikov and Chirikova (2022)
51	Rhodirosin C	C_24_H_20_O_17_	*R. rosea*	Olennikov and Chirikova (2022)
52	Gossypetin 3-*O-β*-D-glucopyranoside	C_21_H_20_O_13_	*R. tangutica*	Olennikov and Prokopyev (2024)
53	Gossypetin 3-*O-β*-D-glucuronopyranoside	C_21_H_18_O_14_	*R. tangutica*	Olennikov and Prokopyev (2024)
54	Gossypetin 8-*O*-*β*-D-xylopyranoside	C_20_H_18_O_12_	*R. rosea*, *R. quadrifida*	Iwashina et al., 2024; Olennikov and Chirikova, 2020
55	Rhodiquadrin D	C_20_H_18_O_12_	*R. quadrifida*	Olennikov and Chirikova (2020)
56	Rhodioflavonoside	C_27_H_30_O_17_	*R. rosea*, *R. wallichiana*, *R. quadrifida*	Zhao et al., 2016; Yoshikawa et al., 1996; Ming et al., 2005
57	Rhodiquadrin A	C_27_H_28_O_19_	*R. quadrifida*	Olennikov and Chirikova (2020)
58	Rhodiolgidin	C_27_H_30_O_17_	*R. rosea*	Olennikov et al. (2020)
59	Gossypetin 3,7-di-*O-β*-D-glucopyranoside	C_27_H_30_O_18_	*R. ishidae*	Iwashina et al. (2023)
60	Gossypetin 3-*O*-*β*-D-glucopyranoside-8-*O-β*-D-glucuronopyranoside	C_27_H_28_O_19_	*R. rosea*, *R. tangutica*, *R. quadrifida*	Olennikov and Prokopyev, 2024; Olennikov and Chirikova, 2020; Olennikov, 2023
61	Rhodiquadrin B	C_29_H_30_O_20_	*R. rosea*, *R. quadrifida*	Olennikov and Chirikova, 2020; Olennikov, 2023
62	Gossypetin 3-*O*-*β*-D-glucuronopyranoside-8-*O-β*-D-glucopyranoside	C_27_H_28_O_19_	*R. rosea*, *R. ishidae*	Iwashina et al., 2024; Iwashina et al., 2023
63	Gossypetin 3-*O*-*β*-D-(2″-malonylxylopyranoside)-8-*O-β*-D-glucopyranoside	C_29_H_30_O_20_	*R. ishidae*	Iwashina et al. (2023)
64	Gossypetin 3,7-di-*O-β*-D-glucopyranoside-8-*O-β*-D-glucuronopyranoside	C_33_H_38_O_24_	*R. rosea*, *R. ishidae*	Iwashina et al., 2023; Olennikov, 2023
65	Kaempferol	C_15_H_10_O_6_	*R. crenulata*, *R. rosea*, *R. kirilowii*, *R. fastigiata*, *R. wallichiana*, *R. tangutica*, *R. sachalinensis*, *R. yunnanensis*, *R. dumulosa*, *R. quadrifida*, *R. sacra*, *R. bupleuroides*, *R. himalensis*, *R. heterodonta*, *R. coccinea*, *R. pamiroalaica*, *R. litwinowii*	Ou et al., 2020; Nan et al., 2018; Zhu et al., 2024; Zhou et al., 2015; Nakamura et al., 2007; Dong et al., 2009; Krasnov et al., 1976; Krasnov and Khoruzhaya, 1974; Ma et al., 2014; Krasnov, 1979; Luo et al., 2005; Hu and Wang, 2022; Jiang et al., 2017; Liu et al., 2000
66	Kaempferol 7-*O*-*α*-L-rhamnopyranoside	C_21_H_20_O_10_	*R. crenulata*, *R. rosea*, *R. wallichiana*, *R. tangutica*, *R. sachalinensis*, *R. dumulosa*, *R. bupleuroides*	Nan et al., 2018; Song et al., 2018; Zhou et al., 2015; Luo et al., 2005; Ni et al., 2013; Dong et al., 2007; Tang et al., 2021
67	Afzelin	C_21_H_20_O_10_	*R. crenulata*, *R. rosea*, *R. tangutica*, *R. sachalinensis*, *R. yunnanensis*, *R. dumulosa*, *R. litwinowii*	Choe et al., 2012; Zhu et al., 2024; Huang H. et al., 2008; Lee et al., 2016; Chen et al., 2024; Krasnov, 1979; Olennikov and Prokopyev, 2024
68	Kaempferol 4′-*O-α*-L-rhamnopyranoside	C_21_H_20_O_10_	*R. crenulata*, *R. sachalinensis*, *R. dumulosa*	Huang H. et al. (2008)
69	Kaempferol 7-*O*-*β*-D-glucopyranoside	C_21_H_20_O_11_	*R. crenulata*, *R. sachalinensis*	Liu et al., 2024a; Ni et al., 2013
70	Astragalin	C_21_H_20_O_11_	*R. crenulata*, *R. rosea*, *R. wallichiana*, *R. sachalinensis*, *R. ishidae*	Ni, 2016; Iwashina et al., 2023; Ni et al., 2016; Wang X. J. et al., 2016; Liu et al., 2020
71	Kaempferol 4′-*O*-*β*-D-glucopyranoside	C_21_H_20_O_11_	*R. tangutica*	Olennikov and Prokopyev (2024)
72	Trifolin	C_21_H_20_O_11_	*R. sachalinensis*	Han et al. (2011)
73	Kaempferol 3-*O-β*-D-sophoroside	C_27_H_30_O_16_	*R. crenulata*, *R. rosea*, *R. sachalinensis*, *R. dumulosa*	Huang H. et al., 2008; Fan et al., 2001; Xu et al., 2013
74	Kaempferol 3-rungioside	C_27_H_30_O_15_	*R. crenulata*, *R. sachalinensis*, *R. dumulosa*	Huang H. et al. (2008)
75	Kaempferol 3-*O-α*-L-rhamnopyranosyl(1→4)-*β*-D-glucopyranoside	C_27_H_30_O_15_	*R. crenulata*, *R. sachalinensis*, *R. dumulosa*	Huang H. et al. (2008)
76	Nicotiflorin	C_27_H_30_O_15_	*R. crenulata*	Zhu et al. (2024)
77	Multiflorin B	C_27_H_30_O_15_	*R. rosea*, *R. sachalinensis*	Choe et al., 2012; Tusungul and Wan, 2018
78	Ternatumoside II	C_27_H_30_O_15_	*R. crenulata*, *R. rosea*, *R. sachalinensis*	Zhou et al., 2015; Zhang et al., 2016
79	Crenuloside	C_27_H_30_O_15_	*R. crenulata*, *R. dumulosa*	Zhou et al., 2015; Wang J. X. et al., 2006
80	Leucoside	C_26_H_28_O_15_	*R. rosea*, *R. wallichiana*, *R. sachalinensis*	Nakamura et al., 2007; Wang X. J. et al., 2016; Ma et al., 2013
81	Kaempferol 3-*O*-*β*-D-xylofuranosyl(1→2)-*β*-D-glucopyranoside	C_26_H_28_O_15_	*R. sachalinensis*	Fan et al. (2001)
82	Kaempferitrin	C_27_H_30_O_14_	*R. rosea*	Olennikov et al. (2020)
83	Kaempferol 3-*O-β*-D-glucopyranoside-7-*O*-*α*-L-rhamnopyranoside	C_27_H_30_O_15_	*R. rosea*, *R. sachalinensis*, *R. dumulosa*, *R. ishidae*	Liu et al., 2008; Iwashina et al., 2023; Ma et al., 2013; Ni et al., 2013
84	Kaempferol 3-*O-α*-L-rhamnopyranoside-7-*O-β*-D-glucopyranoside	C_27_H_30_O_15_	*R. ishidae*	Iwashina et al. (2023)
85	Kaempferol 3,7-di-*O-β*-D-glucopyranoside	C_27_H_30_O_16_	*R. rosea*, *R. ishidae*	Olennikov et al., 2020; Iwashina et al., 2023
86	Kaempferol 3,4′-di-*O-β*-D-glucopyranoside	C_27_H_30_O_16_	*R. sachalinensis*	Choe et al. (2012)
87	Sachaloside III	C_32_H_38_O_19_	*R. sachalinensis*	Nakamura et al. (2007)
88	Kaempferol 4′-*O-α*-L-rhamnopyranoside-3-*O-β*-D-xylopyranosyl(1→2)-*β*-D-glucopyranoside	C_32_H_38_O_19_	*R. rosea*	Liu et al. (2020)
89	Kaempferol 3-*O-α*-L-rhamnopyranoside-7-*O-β*-D-sophoroside	C_33_H_40_O_20_	*R. ishidae*	Iwashina et al. (2023)
90	Kaempferol 3-*O-β*-D-sophoroside-7-*O-β*-D-glucopyranoside	C_33_H_40_O_21_	*R. ishidae*	Iwashina et al. (2023)
91	3,3′,5,7-Tetrahydroxyflavone	C_15_H_10_O_6_	*R. crenulata*	Ni et al. (2016)
92	Quercetin	C_15_H_10_O_7_	*R. crenulata*, *R. rosea*, *R. kirilowii*, *R. fastigiata*, *R. wallichiana*, *R. tangutica*, *R. sachalinensis*, *R. dumulosa*, *R. quadrifida*, *R. sacra*, *R. bupleuroides*, *R. himalensis*, *R. heterodonta*, *R. coccinea*, *R. atuntsuensis*	Nan et al., 2018; Chen et al., 1999; Zhu et al., 2024; Chen et al., 2024; Krasnov et al., 1976; Polumackanycz et al., 2022; Krasnov and Khoruzhaya, 1974; Liu et al., 2008; Wang X. J. et al., 2016; Hu and Wang, 2022; Dong et al., 2007
93	Tamarixetin	C_16_H_12_O_7_	*R. sachalinensis*	Ni et al. (2013)
94	Quercetin 3′,4′,7-trimethyl ether	C_18_H_16_O_7_	*R. crenulata*	Ni et al. (2016)
95	Quercitrin	C_21_H_20_O_11_	*R. crenulata*, *R. rosea*, *R. wallichiana*, *R. tangutica*, *R. sacra*, *R. himalensis*	Ou et al., 2020; Zhu et al., 2024; Olennikov et al., 2020; Chen et al., 2024; Olennikov and Prokopyev, 2024
96	Quercetin 7-*O-β*-D-glucopyranoside	C_21_H_20_O_12_	*R. bupleuroides*, *R. ishidae*	Wang H. et al., 2016; Iwashina et al., 2023
97	Isoquercitrin	C_21_H_20_O_12_	*R. crenulata*, *R. rosea*, *R. wallichiana*, *R. tangutica*, *R. sachalinensis*, *R. sacra*, *R. heterodonta*, *R. coccinea*, *R. ishidae*	Ou et al., 2020; Nan et al., 2018; Zhu et al., 2024; Chen et al., 2024; Krasnov et al., 1976; Krasnov and Khoruzhaya, 1974; Iwashina et al., 2023; Olennikov, 2023; Ni et al., 2013
98	Quercetin 3-*O*-*β*-D-glucuronopyranoside	C_21_H_18_O_13_	*R. crenulata*	Chen et al. (2024)
99	Hyperoside	C_21_H_20_O_12_	*R. crenulata*, *R. coccinea*	Krasnov and Khoruzhaya, 1974; Ren et al., 2021
100	Rutin	C_27_H_30_O_16_	*R. crenulata*, *R. rosea*, *R. kirilowii*, *R. fastigiata*, *R. wallichiana*, *R. sachalinensis*, *R. dumulosa*, *R. atuntsuensis*	Chen et al., 1999; Song et al., 2018; Polumackanycz et al., 2022; Ding et al., 2018; Rei et al., 2021
101	Baimaside	C_27_H_30_O_17_	*R. ishidae*	Iwashina et al. (2023)
102	Quercetin 3-*O-β*-D-glucopyranoside-7-*O-α*-L-rhamnopyranoside	C_27_H_30_O_16_	*R. rosea*	Olennikov et al. (2020)
103	Quercetin 3,7-di-*O-β*-D-glucopyranoside	C_27_H_30_O_17_	*R. rosea*, *R. ishidae*	Olennikov et al., 2020; Iwashina et al., 2023
104	Myricetin 3-*O*-*β*-D-glucopyranoside	C_21_H_20_O_13_	*R. ishidae*	Iwashina et al. (2023)
105	Hibiscetin 3-*O*-*β*-D-glucopyranoside-8-*O-β*-D-glucuronopyranoside	C_27_H_28_O_20_	*R. rosea*, *R. tangutica*	Iwashina et al., 2024; Olennikov and Prokopyev, 2024
106	Rhodirosin G	C_30_H_30_O_23_	*R. rosea*	Olennikov (2023)
107	Rhodirosin H	C_30_H_30_O_23_	*R. rosea*	Olennikov (2023)
108	Apigenin	C_15_H_10_O_5_	*R. sacra*	Jiang et al. (2017)
109	Apigenin 7-*O-β*-D-apiofuranosyl(1→2)-*β*-D-glucopyranoside	C_26_H_28_O_14_	*R. tangutica*	Zhang et al. (2010)
110	Vicenin-2	C_27_H_30_O_15_	*R. ishidae*	Iwashina et al. (2023)
111	Luteolin	C_15_H_10_O_6_	*R. crenulata*, *R. rosea*, *R. kirilowii*, *R. fastigiata*, *R. wallichiana*, *R. sachalinensis*, *R. yunnanensis*, *R. sacra*, *R. himalensis*	Zuo et al., 2007; Zhu et al., 2024; Zhou et al., 2015; Nakamura et al., 2007; Iwashina et al., 2024
112	Luteolin 7-ethyl ether	C_17_H_14_O_6_	*R. fastigiata*	Yang et al. (2002)
113	Cynaroside	C_21_H_20_O_11_	*R. crenulata*, *R. fastigiata*, *R. sachalinensis*	Li et al., 2021; Chen et al., 2024; Ding et al., 2018
114	Luteolin 3′,7-dimethyl ether	C_17_H_14_O_6_	*R. crenulata*	Ni et al. (2016)
115	3′-Methoxyl luteolin 7-*O*-*β*-D-glucoside	C_22_H_22_O_11_	*R. sachalinensis*	Ding et al. (2018)
116	Lucenin-2	C_27_H_30_O_16_	*R. ishidae*	Iwashina et al. (2023)
117	Tricetin	C_15_H_10_O_7_	*R. kirilowii*, *R. quadrifida*	Zuo et al., 2007; Yoshikawa et al., 1996
118	Tricin	C_17_H_14_O_7_	*R. crenulata*, *R. rosea*, *R. wallichiana*, *R. sachalinensis*, *R. sacra*, *R. henryi*, *R. himalensis*	Daikonya and Kitanaka, 2011; Nakamura et al., 2007; Ni et al., 2016; Wang X. J. et al., 2016; Masi et al., 2023; Lou and Zuo, 1990; Qiu et al., 1989
119	Tricin 5-*O*-*β*-D-glucopyranoside	C_23_H_24_O_12_	*R. rosea*	Langeder et al. (2021)
120	Tricin 7-*O*-*β*-D-glucopyranoside	C_23_H_24_O_12_	*R. crenulata*, *R. rosea*, *R. henryi*	Ni et al., 2016; Lou and Zuo, 1990; Kurkin et al., 1982
121	Dihydrokaempferol	C_15_H_12_O_6_	*R. rosea*, *R. fastigiata*, *R. sacra*	Lee et al., 2016; Daikonya and Kitanaka, 2011; Peng et al., 1996
122	Naringenin	C_15_H_12_O_5_	*R. sacra*	Daikonya and Kitanaka (2011)
123	Naringenin 7-*O-β*-D-sophoroside	C_27_H_32_O_15_	*R. ishidae*	Iwashina et al. (2023)
124	Eriodictyol	C_15_H_12_O_6_	*R. crenulata*, *R. fastigiata*, *R. tangutica*, *R. sachalinensis*, *R. yunnanensis*, *R. sacra*, *R. himalensis*	Nan et al., 2018; Zhu et al., 2024; Nakamura et al., 2007; Chen et al., 2024
125	5,7,3′,5′-Tetrahydroxyflavanone	C_15_H_12_O_6_	*R. crenulata*, *R. rosea*, *R. sacra*	Daikonya and Kitanaka, 2011; Zhou et al., 2015; Wang et al., 2010
126	Genistein	C_15_H_10_O_5_	*R. crenulata*	Zhao (2015)
127	Phloretin	C_15_H_14_O_5_	*R. crenulata*	Liu et al. (2024a)
128	Cyanidin 3-*O-β*-D-glucopyranoside	C_21_H_21_O_11_^+^	*R. rosea*, *R. ishidae*	Iwashina et al., 2024; Iwashina et al., 2023
129	(+)-Catechin	C_15_H_14_O_6_	*R. crenulata*, *R. rosea*, *R. kirilowii*, *R. fastigiata*, *R. wallichiana*, *R. tangutica*, *R. sacra*, *R. bupleuroides*, *R. himalensis*, *R. coccinea*, *R. alsia*, *R. semenovii*, *R. chrysanthemifolia*, *R. sexifolia*, *R. tieghemii*	Ma et al., 2022; Liu et al., 2013; Ou et al., 2020; Nan et al., 2018; Chi et al., 2012; Zhu et al., 2024; Polumackanycz et al., 2022; Gryszczyńska et al., 2012; Kuliev et al., 2004
130	(−)-Catechin gallate(CG)	C_22_H_18_O_10_	*R. crenulata*, *R. rosea*, *R. sacra*	Zhu et al., 2024; Olennikov et al., 2020; Chen et al., 2024
131	(−)-Epicatechin(EC)	C_15_H_14_O_6_	*R. crenulata*, *R. rosea*, *R. kirilowii*, *R. fastigiata*, *R. wallichiana*, *R. tangutica*, *R. yunnanensis*, *R. sacra*, *R. bupleuroides*, *R. himalensis*, *R. coccinea*, *R. alsia*, *R. semenovii*, *R. chrysanthemifolia*, *R. sexifolia*, *R. tieghemii*	Ou et al., 2020; Nan et al., 2018; Chi et al., 2012; Zhu et al., 2024; Guo et al., 1993; Gryszczyńska et al., 2012; Kuliev et al., 2004; Chu et al., 2014a
132	(−)-Epicatechin gallate(ECG)	C_22_H_18_O_10_	*R. crenulata*, *R. rosea*, *R. kirilowii*, *R. fastigiata*, *R. wallichiana*, *R. tangutica*, *R. yunnanensis*, *R. sacra*, *R. semenovii*	Ou et al., 2020; Nan et al., 2018; Zhu et al., 2024; Li et al., 2021; Gryszczyńska et al., 2012; Kuliev et al., 2004; Chu et al., 2014a
133	(+)-Gallocatechin	C_15_H_14_O_7_	*R. semenovii*	Kuliev et al. (2004)
134	(−)-Gallocatechin	C_15_H_14_O_7_	*R. rosea*	Olennikov et al. (2020)
135	(−)-Gallocatechin gallate	C_22_H_18_O_11_	*R. rosea*	Olennikov et al. (2020)
136	(−)-Epigallocatechin(EGC)	C_15_H_14_O_7_	*R. rosea*, *R. kirilowii*, *R. sachalinensis*, *R. semenovii*, *R. recticaulis*	Olennikov et al., 2019; Fan et al., 2001; Gryszczyńska et al., 2012; Kuliev et al., 2004
137	(−)-Epigallocatechin gallate(EGCG)	C_22_H_18_O_11_	*R. crenulata*, *R. rosea*, *R. kirilowii*, *R. fastigiata*, *R. yunnanensis*, *R. sachalinensis*, *R. sacra*, *R. heterodonta*, *R. gelida*, *R. litwinowii*, *R. semenovii*, *R. recticaulis*	Olennikov et al., 2019; Fan et al., 1999; Li et al., 2021; Guo et al., 1993; Yang et al., 2012a; Lee et al., 2000; Gryszczyńska et al., 2012; Zhang et al., 2022; Melikuziev et al., 2013; Yousef et al., 2006
138	Procyanidin A1	C_30_H_24_O_12_	*R. crenulata*	Jia et al. (2024)
139	Procyanidin B1	C_30_H_26_O_12_	*R. crenulata*	Ma et al. (2022)
140	Procyanidin B2	C_30_H_26_O_12_	*R. crenulata*	Jia et al. (2024)
141	Procyanidin B2 3″-*O*-gallate	C_37_H_30_O_16_	*R. crenulata*, *R. semenovii*	Kuliev et al., 2004; Chu et al., 2014b
142	Procyanidin B2 3,3′-di-*O*-gallate	C_44_H_34_O_20_	*R. kirilowii*, *R. semenovii*	Zuo et al., 2007; Kuliev et al., 2004
143	Procyanidin B3	C_30_H_26_O_12_	*R. crenulata*, *R. semenovii*	Chen et al., 2021; Kuliev et al., 2004
144	(−)-Epicatechin-(4β→8)-(−)-epigallocatechin 3-*O*-gallate	C_37_H_30_O_17_	*R. semenovii*	Kuliev (2010)
145	3′-Galloylprodelphinidin B2	C_37_H_30_O_18_	*R. heterodonta*, *R. recticaulis*	Olennikov et al., 2019; Grace et al., 2009
146	Rhodisin	C_44_H_34_O_22_	*R. kirilowii*, *R. sachalinensis*, *R. sacra*, *R. heterodonta*, *R. gelida*, *R. recticaulis*	Zuo et al., 2007; Olennikov et al., 2019; Grace et al., 2009; Fan et al., 2001; Fan et al., 1999
147	3-*O*-Galloylepigallocatechin-(4*α*→8)-epigallocatechin 3-*O*-gallate	C_44_H_34_O_22_	*R. rosea*	van Diermen et al. (2009)
148	Procyanidin C1	C_45_H_38_O_18_	*R. crenulata*	Jia et al. (2024)
149	Rhodikhinoside	C_95_H_80_O_48_	*R. semenovii*	Khi et al. (1991)
150	Rhodikhim	C_88_H_66_O_38_	*R. semenovii*	Khi et al. (1991)
151	Proanthocyanidin CS-1	C_121_H_118_O_65_	*R. semenovii*	Matamarova et al. (1998)
152	Proanthocyanidin CS-2	C_113_H_100_O_62_	*R. semenovii*	Matamarova et al. (1998)
153	Proanthocyanidin CS-3	C_127_H_128_O_69_	*R. semenovii*	Matamarova et al. (1999)
154	Proanthocyanidin CS-4	C_136_H_120_O_70_	*R. semenovii*	Matamarova et al. (1999)
155	Rhodichimoside	C_112_H_106_O_64_	*R. semenovii*	Kuliev et al. (2000)
156	Rhodichin	C_95_H_80_O_48_	*R. semenovii*	Kuliev et al. (2000)
157	Proanthocyanidin RP-1	C_125_H_130_O_69_	*R. pamiroalaica*	Ismailov et al. (1998)
158	Proanthocyanidin RP-2	C_120_H_114_O_64_	*R. pamiroalaica*	Ismailov et al. (1998)
159	Proanthocyanidin RP-3	C_136_H_120_O_70_	*R. pamiroalaica*	Ismailov et al. (1999)
160	Proanthocyanidin RP-4	C_129_H_106_O_67_	*R. pamiroalaica*	Ismailov et al. (1999)

Flavonols (1–107) dominate the flavonoid profile, featuring aglycone cores of herbacetin, gossypetin, kaempferol, and quercetin. These compounds exist mainly as glycosides substituted at the C-3, C-7, C-8, or C-4′ positions, with sugar moieties including rhamnose, glucose, glucuronic acid, arabinose, xylose, and their acetylated derivatives. Among these, herbacetin derivatives are the most representative constituents, with rhodionin and rhodiosin being the most extensively reported. Glucuronides, with herbacetin and gossypetin as their principal aglycones, show preferential accumulation in aerial parts. A structurally unique metabolite, rhodiolin, results from the condensation of dihydroconiferyl alcohol (a phenylpropanoid) with herbacetin (a flavonol) and has been consistently detected across multiple *Rhodiola* species.

The second predominant class is flavan-3-ols (129–137), such as (+)-catechin, (−)-epicatechin, (−)-epicatechin gallate, (−)-epigallocatechin, and (−)-epigallocatechin gallate. These compounds also serve as building blocks that polymerize via C4-C8 or C4-C6 bonds to form various oligomers and polymers, collectively known as proanthocyanidins (138–160).

Additionally, *Rhodiola* species contain a small number of other flavonoid subclasses, including flavones (108–120; mainly luteolin and tricin derivatives), flavanones (121–125), isoflavone (126), dihydrochalcone (127), and anthocyanidin (128).

### 3.4 Terpenoids

**FIGURE 4:**
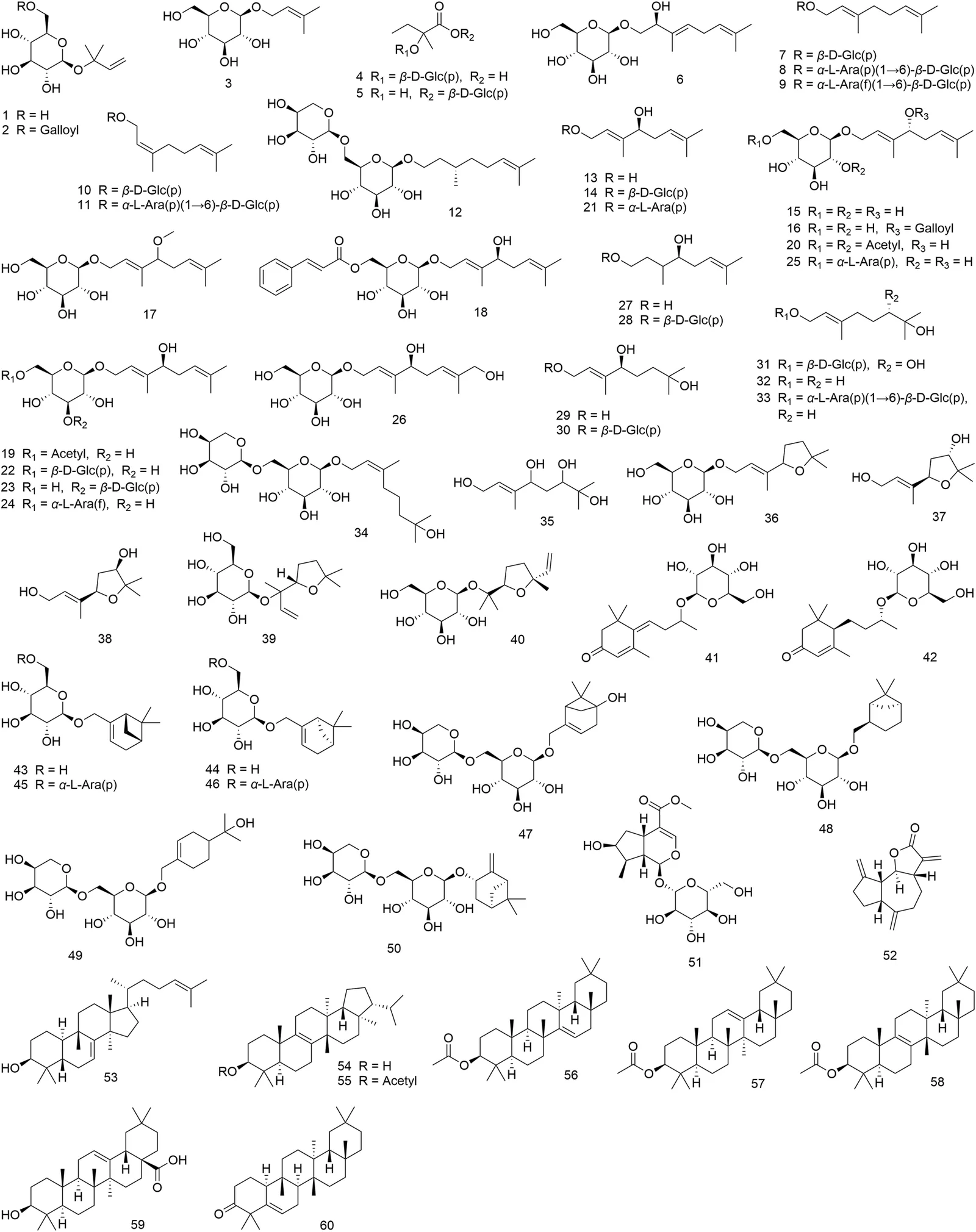
Structures of terpenoids.

Terpenoids also play a significant role in the phytochemical profile of *Rhodiola* species. Up to now, approximately 60 terpenoid compounds have been isolated according to our investigations, comprising hemiterpenoids (1–5), monoterpenoids (6–51), a sesquiterpenoid (52), and triterpenoids (53–60). The details of these compounds are presented in Table 5 and Figure 4.

**TABLE 5:** Terpenoids from *Rhodiola* species.

No.	Compounds	Molecular formula	Source of species	References
1	Crenulatin	C_11_H_20_O_6_	*R. crenulata*, *R. rosea*, *R. kirilowii*, *R. fastigiata*, *R. wallichiana*, *R. yunnanensis*, *R. sacra*, *R. himalensis*	Zhu et al., 2024; Lee et al., 2016; Nakamura et al., 2008; Zhang, 2021; Yoshikawa et al., 1997
2	Taxilluside A	C_18_H_24_O_10_	*R. crenulata*	Liu et al. (2024a)
3	3-Methyl-but-2-en-1-yl-*O*-*β*-D-glucopyranoside	C_11_H_20_O_6_	*R. crenulata*, *R. rosea*	Lee et al., 2016; Liu et al., 2024a
4	2-Methyl butyric acid-2-*O-β*-D-glucopyranoside	C_11_H_20_O_8_	*R. wallichiana*	Chai (2015)
5	1-(2-Hydroxy-2-methylbutanoate)-*β*-D-glucopyranose	C_11_H_20_O_8_	*R. kirilowii*	Yang et al. (2011a)
6	(3*E*,2*S*)-2-Hydroxy-3,7-dimethyl-3,6-octadien-1-yl *β*-D-glucopyranoside	C_16_H_28_O_7_	*R. rosea*	Tang et al. (2023)
7	Geranyl 1-*O-β*-D-glucopyranoside	C_16_H_28_O_6_	*R. rosea*, *R. kirilowii*, *R. sachalinensis*	Nakamura et al., 2007; Huang Y. J. et al., 2008; Tang et al., 2021
8	Kenposide A	C_21_H_36_O_10_	*R. crenulata*, *R. rosea*, *R. sachalinensis*, *R. sacra*	Nakamura et al., 2007; Nakamura et al., 2008; Mudge et al., 2012; Yoshikawa et al., 1997
9	Geranyl 1-*O-α*-L-arabinofuranosyl(1→6)-*β*-D-glucopyranoside	C_21_H_36_O_10_	*R. rosea*, *R. sachalinensis*	Nakamura et al., 2007; Ali et al., 2008
10	Neryl 1-*O-β*-D-glucopyranoside	C_16_H_28_O_6_	*R. kirilowii*	Huang Y. J. et al. (2008)
11	Sacranoside B	C_21_H_36_O_10_	*R. kirilowii*, *R. sacra*	Huang Y. J. et al., 2008; Yoshikawa et al., 1997
12	Creoside V	C_21_H_38_O_10_	*R. crenulata*	Nakamura et al. (2008)
13	(−)-Rosiridol	C_10_H_18_O_2_	*R. rosea*, *R. linearifolia*	Kurlin and Zapesochnaya, 1986; Masi et al., 2023
14	(4*S*)-Rosiridin	C_16_H_28_O_7_	*R. crenulata*, *R. rosea*, *R. wallichiana*, *R. sachalinensis*, *R. tibetica*, *R. linearifolia*, *R. recticaulis*	Ma et al., 2008; Olennikov et al., 2019; Han et al., 2016; Liu et al., 2019; Kurlin and Zapesochnaya, 1986; Tang et al., 2023; Yoshikawa et al., 2008
15	(4*R*)-Rosiridin	C_16_H_28_O_7_	*R. rosea*, *R. sachalinensis*	Fan et al., 2001; Tang et al., 2023
16	Sachalinoside A	C_23_H_32_O_11_	*R. sachalinensis*	Fan et al. (2001)
17	(2*E*)-4-Methoxy-3,7-dimethyl-2,6-octadien-1-yl *β*-D-glucopyranoside	C_17_H_30_O_7_	*R. rosea*	Tang et al. (2023)
18	Rhodiosidin	C_25_H_34_O_8_	*R. rosea*	Masi et al. (2023)
19	Rosiridoside C	C_18_H_30_O_8_	*R. rosea*, *R. sachalinensis*	Olennikov and Chirikova, 2024; Yoshikawa et al., 2008
20	Rhodirosin N	C_20_H_32_O_9_	*R. rosea*	Olennikov and Chirikova (2024)
21	Rosiridoside A	C_15_H_26_O_6_	*R. sachalinensis*	Yoshikawa et al. (2008)
22	Rhodioloside B	C_22_H_38_O_12_	*R. rosea*	Ma et al., 2006; Li W. et al., 2008
23	Rhodioloside C	C_22_H_38_O_12_	*R. rosea*	Ma et al., 2006; Li W. et al., 2008
24	Rosiridoside B	C_21_H_36_O_11_	*R. sachalinensis*	Yoshikawa et al. (2008)
25	Rhodioloside F	C_21_H_36_O_11_	*R. rosea*, *R. sachalinensis*	Ali et al., 2008; Avula et al., 2009
26	Rhodioloside A	C_16_H_28_O_8_	*R. rosea*	Ma et al., 2006; Li W. et al., 2008
27	Sachalol	C_10_H_20_O_2_	*R. sachalinensis*	Li X. Z. et al. (2008)
28	Sachaloside VI	C_16_H_30_O_7_	*R. sachalinensis*	Li X. Z. et al. (2008)
29	Sachalinol A	C_10_H_20_O_3_	*R. rosea*, *R. sachalinensis*	Fan et al., 2001; Ali et al., 2008
30	Rhodioloside D	C_16_H_30_O_8_	*R. rosea*, *R. sachalinensis*	Avula et al., 2009; Ma et al., 2006; Li W. et al., 2008
31	Sachaloside VII	C_16_H_30_O_8_	*R. sachalinensis*	Li X. Z. et al. (2008)
32	(*E*)-3,7-Dimethyl-2-octene-1,7-diol	C_10_H_20_O_2_	*R. rosea*	Tang et al. (2021)
33	Rhodioloside E /Sachaloside VIII	C_21_H_38_O_11_	*R. crenulata*, *R. rosea*, *R. sachalinensis*, *R. sacra*	Daikonya and Kitanaka, 2011; Nakamura et al., 2008; Ma et al., 2006; Li X. Z. et al., 2008
34	Rhodioloside G	C_21_H_38_O_11_	*R. sacra*	Daikonya and Kitanaka (2011)
35	1,4,6,7-Tetrahydroxy-3,7-dimethyl-2-octene	C_10_H_20_O_4_	*R. rosea*	Ma (2006)
36	3-(5,5-Dimethyltetrahydrofuranyl)-2*E*-buten-1-yl *β*-D-glucopyranoside	C_16_H_28_O_7_	*R. rosea*	Tang et al. (2023)
37	Sachalinol B	C_10_H_18_O_3_	*R. sachalinensis*	Fan et al. (2001)
38	Sachalinol C	C_10_H_18_O_3_	*R. sachalinensis*	Fan et al. (2001)
39	Sachalinoside B	C_16_H_28_O_7_	*R. sachalinensis*	Fan et al. (2001)
40	*trans*-Linalool-3,6-oxide-*β*-D-glucopyranoside	C_16_H_28_O_7_	*R. crenulata*	Liu et al. (2024a)
41	(6*Z*)-4-(3-Hydroxybutylidene)-3,5,5-trimethyl-2-cyclohexene-1-one-*O*-*β*-D-glucopyranoside	C_19_H_30_O_7_	*R. crenulata*	Liu et al. (2024a)
42	Blumenyl C *β*-D-glucopyranoside	C_19_H_32_O_7_	*R. crenulata*	Liu et al. (2024a)
43	(+)-Myrtenyl *β*-D-glucopyranoside	C_16_H_26_O_6_	*R. rosea*	Tang et al. (2020)
44	(−)-Myrtenyl *β*-D-glucopyranoside	C_16_H_26_O_6_	*R. rosea*, *R. sachalinensis*	Nakamura et al., 2007; Tang et al., 2020
45	(+)-Myrtenyl *α*-L-arabinopyranosyl(1→6)-*β*-D-glucopyranoside	C_21_H_34_O_10_	*R. rosea*	Tang et al. (2020)
46	Sacranoside A	C_21_H_34_O_10_	*R. rosea*, *R. sachalinensis*, *R. sacra*	Nakamura et al., 2007; Yoshikawa et al., 1997; Tang et al., 2020
47	Sacranoside C	C_21_H_34_O_11_	*R. sacra*	Daikonya and Kitanaka (2011)
48	Sachaloside I	C_21_H_36_O_10_	*R. sachalinensis*	Nakamura et al. (2007)
49	Sacranoside D	C_21_H_36_O_11_	*R. sacra*	Daikonya and Kitanaka (2011)
50	Sachaloside II	C_21_H_34_O_10_	*R. rosea*, *R. sachalinensis*	Nakamura et al., 2007; Tang et al., 2021
51	Loganin	C_17_H_26_O_10_	*R. crenulata*	Liu et al. (2024a)
52	Dehydrocostus lactone	C_15_H_18_O_2_	*R. fastigiata*	Gou et al. (2024)
53	10*α*-Cucurbita-7,24-diene-3*β*-ol	C_30_H_50_O	*R. fastigiata*	Gou et al. (2024)
54	Isomotiol	C_30_H_50_O	*R. sachalinensis*	Li et al. (1998)
55	Isomotiyl acetate	C_32_H_52_O_2_	*R. henryi*	Lou and Zuo (1991)
56	Taraxeryl acetate	C_32_H_52_O_2_	*R. sachalinensis*, *R. henryi*	Lou and Zuo, 1991; Li et al., 1998
57	*β*-Amyrin acetate	C_32_H_52_O_2_	*R. fastigiata*	Gou et al. (2024)
58	Isomultiflorenyl acetate	C_32_H_52_O_2_	*R. henryi*	Lou and Zuo (1991)
59	Oleanic acid	C_30_H_48_O_3_	*R. linearifolia*	Kurlin and Zapesochnaya (1986)
60	Glutinone	C_30_H_48_O	*R. pamiroalaica*	Liu et al. (2000)

Hemiterpenoids are relatively rare in nature and often chemically unstable, particularly in their glycosylated forms. Only five hemiterpenoid glycosides have been discovered in the genus, among which crenulatin is the most representative and has been reported in multiple *Rhodiola* species.

Monoterpenoids and their glycosides dominate the terpenoid profile in *Rhodiola* species, with rosiridin being the most representative. However, the absolute configuration of rosiridin and structurally related C-4 hydroxylated geraniol derivatives has long been debated. Recent work by Tang et al. resolved this controversy through NMR-based Mosher ester analysis, confirming the enantiomeric nature of rosiridin’s aglycone moiety (Tang et al., 2023).

### 3.5 Phenolics and organic acids

**FIGURE 5:**
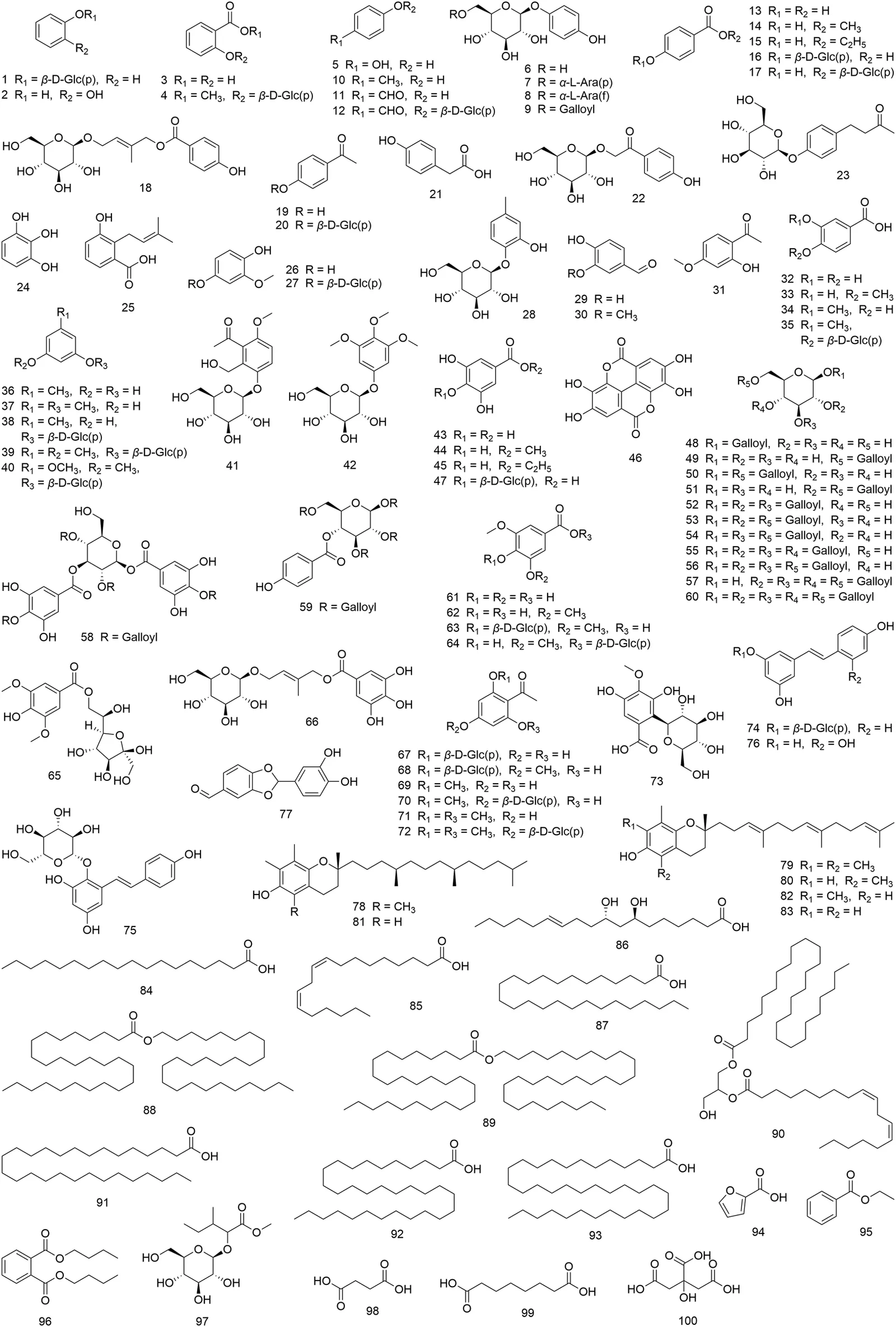
Structures of phenolics and organic acids.

*Rhodiola* species are rich sources of diverse phenolic and organic acid compounds. The dominant compounds in this class are phenolics and phenolic acids (1–83), with arbutin and gallic acid being the representative constituents. Additionally, a limited number of fatty acids (84–93) and several other organic acids (94–100) have now been isolated from *Rhodiola* species. The details of these compounds are presented in Table 6 and Figure 5.

**TABLE 6:** Phenolics and organic acids from *Rhodiola* species.

No.	Compounds	Molecular formula	Source of species	References
1	Phenyl-*β*-D-glucopyranoside	C_12_H_16_O_6_	*R. imbricata*	Choudhary et al. (2015a)
2	Catechol	C_6_H_6_O_2_	Uncertain	Zhou et al. (2007)
3	Salicylic acid	C_7_H_6_O_3_	Uncertain	Zhou et al. (2007)
4	Methyl-2-*O*-*β*-D-glucopyranosylbenzoate	C_14_H_18_O_8_	*R. rosea*	Liu et al. (2024b)
5	Hydroquinone	C_6_H_6_O_2_	*R. crenulata*, *R. rosea*, *R. sacra*, *R. coccinea*	Fan et al., 1999; Chen, 2009; Wang H. et al., 2007; Khoruzhaya and Krasnov, 1972
6	Arbutin	C_12_H_16_O_7_	*R. crenulata*, *R. rosea*, *R. kirilowii*, *R. fastigiata*, *R. wallichiana*, *R. tangutica*, *R. sachalinensis*, *R. sacra*, *R. gelida*, *R. coccinea*, *R. recticaulis*	Nan et al., 2018; Olennikov et al., 2019; Olennikov and Chirikova, 2024; Fan et al., 1999; Olennikov et al., 2020; Wang Y. S. et al., 2020; Hu and Wang, 2022; Khoruzhaya and Krasnov, 1972; Qi et al., 2024
7	Rhodirosin I	C_17_H_24_O_11_	*R. rosea*	Olennikov and Chirikova (2024)
8	Rhodirosin J	C_17_H_24_O_11_	*R. rosea*	Olennikov and Chirikova (2024)
9	6′-*O*-Galloyl arbutin	C_19_H_20_O_11_	*R. coccinea*	Krasnov et al. (1975)
10	*p*-Cresol	C_7_H_8_O	*R. crenulata*	Qu et al. (2020)
11	4-Hydroxybenzaldehyde	C_7_H_6_O_2_	*R. kirilowii*, *R. imbricata*	Choudhary et al., 2015a; Yang et al., 2011a
12	Helicid	C_13_H_16_O_7_	*R. rosea*	Liu et al. (2024b)
13	4-Hydroxybenzoic acid	C_7_H_6_O_3_	*R. crenulata*, *R. rosea*, *R. kirilowii*, *R. wallichiana*, *R. sachalinensis*, *R. sacra*, *R. gelida*	Ou et al., 2020; Olennikov et al., 2019; Daikonya and Kitanaka, 2011; Yang et al., 2011a; Liu et al., 2024b; Yang et al., 2012a; Ni et al., 2013
14	Methyl 4-hydroxybenzoate	C_8_H_8_O_3_	*R. rosea*	Lee et al. (2016)
15	Ethylparaben	C_9_H_10_O_3_	*R. kirilowii*	Yang et al. (2011a)
16	4-Hydroxybenzoic acid 4-*O*-*β*-D-glucopyranoside	C_13_H_16_O_8_	*R. crenulata*, *R. gelida*	Olennikov et al., 2019; Yang et al., 2012a
17	1-*O*-(4-Hydroxybenzoyl)-*β*-D-glucopyranose	C_13_H_16_O_8_	*R. sacra*	Daikonya and Kitanaka (2011)
18	Creoside III	C_18_H_24_O_9_	*R. crenulata*, *R. rosea*	Lee et al., 2016; Nakamura et al., 2008
19	*p*-Hydroxyacetophenone	C_8_H_8_O_2_	*R. crenulata*, *R. kirilowii*, *R. gelida*, *R. pamiroalaica*, *R. imbricata*, *R. litwinowii*	Choudhary et al., 2015a; Yang et al., 2011a; Krasnov and Alekseyuk, 1979; Liu et al., 2000; Chu et al., 2014b; Krasnov et al., 1973
20	Picein	C_14_H_18_O_7_	*R. crenulata*, *R. rosea*, *R. wallichiana*, *R. gelida*, *R. pamiroalaica*, *R. litwinowii*, *R. recticaulis*	Chen D. J. et al., 2012; Olennikov et al., 2019; Wang et al., 2015; Tolonen et al., 2003; Liu et al., 2000; Krasnov et al., 1973
21	4-Hydroxyphenylacetic acid	C_8_H_8_O_3_	*R. rosea*, *R. wallichiana*	Wang C. H. et al., 2020; Ou et al., 2020
22	*p*-Hydroxyphenacyl-*β*-D-glucopyranoside	C_14_H_18_O_8_	*R. crenulata*	Chen D. J. et al. (2012)
23	Raspberry ketone glucoside	C_16_H_22_O_7_	*R. rosea*	Ma et al. (2012)
24	Pyrogallol	C_6_H_6_O_3_	*R. crenulata*, *R. wallichiana*, *R. linearifolia*	Song et al., 2018; Kurlin and Zapesochnaya, 1986; Wang and Wang, 1992
25	3-Hydroxy-2-(3-methyl-2-buten-1-yl)-benzoic acid	C_12_H_14_O_3_	*R. imbricata*	Choudhary et al. (2015a)
26	2-Methoxyhydroquinone	C_7_H_8_O_3_	*R. sachalinensis*	Ding (2019)
27	Tachioside	C_13_H_18_O_8_	*R. crenulata*	Yang et al. (2013)
28	2-Hydroxy-4-methylphenyl-*β*-D-glucopyranoside	C_13_H_18_O_7_	*R. imbricata*	Choudhary et al. (2015a)
29	Protocatechuic aldehyde	C_7_H_6_O_3_	*R. crenulata*	Chen et al. (2024)
30	Vanillin	C_8_H_8_O_3_	*R. crenulata*, *R. wallichiana*	Zhao, 2016; Sun et al., 2020
31	Paeonol	C_9_H_10_O_3_	*R. rosea*, *R. wallichiana*	Zhao, 2016; Liu et al., 2024b
32	Protocatechuic acid	C_7_H_6_O_4_	*R. crenulata*, *R. rosea*, *R. fastigiata*, *R. wallichiana*, *R. sachalinensis*, *R. sacra*	Song et al., 2018; Fan et al., 1999; Li et al., 2021; Yang et al., 2012a; Ding, 2019; Wglarz et al., 2008
33	Isovanillic acid	C_8_H_8_O_4_	*R. wallichiana*	Zhao et al. (2016)
34	Vanillic acid	C_8_H_8_O_4_	*R. crenulata*, *R. rosea*, *R. wallichiana*, *R. tangutica*	Nan et al., 2018; Song et al., 2018; Polumackanycz et al., 2022; Yang et al., 2012a
35	Vanillic acid 4-*O*-*β*-D-glucopyranoside	C_14_H_15_O_9_	*R. crenulata*	Yang et al. (2012a)
36	Orcinol	C_7_H_8_O_2_	*R. imbricata*	Choudhary et al. (2015a)
37	3-Methyl-5-methoxyphenol	C_8_H_10_O_2_	*R. imbricata*	Choudhary et al. (2015a)
38	3-Methyl-5-hydroxyphenyl-*β*-D-glucopyranoside	C_13_H_18_O_7_	*R. imbricata*	Choudhary et al. (2015a)
39	3-Methyl-5-methoxyphenyl-*β*-D-glucopyranoside	C_14_H_20_O_7_	*R. imbricata*	Choudhary et al. (2015a)
40	3,5-Dimethoxyphenyl-*β*-D-glucopyranoside	C_14_H_20_O_8_	*R. imbricata*	Choudhary et al. (2015a)
41	2-Hydroxymethyl-3-acetyl-4-methoxyphenyl-*β*-D-glucopyranoside	C_16_H_22_O_9_	*R. imbricata*	Choudhary et al., 2015a; Choudhary et al., 2015b
42	3,4,5-Trimethoxyphenyl-*β*-D-glucopyranoside	C_15_H_22_O_9_	*R. imbricata*	Choudhary et al. (2015a)
43	Gallic acid	C_7_H_6_O_5_	*R. crenulata*, *R. rosea*, *R. kirilowii*, *R. fastigiata*, *R. wallichiana*, *R. tangutica*, *R. sachalinensis*, *R. yunnanensis*, *R. dumulosa*, *R. quadrifida*, *R. sacra*, *R. bupleuroides*, *R. henryi*, *R. himalensis*, *R. heterodonta*, *R. gelida*, *R. coccinea*, *R. alsia*, *R. atuntsuensis*, *R. linearifolia*, *R. purpureoviridis*, *R. pamiroalaica*, *R. imbricata*, *R. litwinowii*, *R. semenovii*, *R. subopposita*, *R. chrysanthemifolia*, *R. sexifolia*, *R. tieghemii*, *R. serrata*, *R. recticaulis*	Liu et al., 2013; Wang C. H. et al., 2020; Zuo et al., 2007; Ou et al., 2020; Chi et al., 2012; Olennikov et al., 2019; Chen et al., 1999; Ma et al., 1995; Rattan et al., 2020; Zhu et al., 2024; Daikonya and Kitanaka, 2011; Fan et al., 2001; Krasnov et al., 1976; Kurlin and Zapesochnaya, 1986; Yang et al., 2012a; Wang H. L. et al., 2006; Olennikov and Prokopyev, 2024; Liu et al., 2000; Rei et al., 2021; Lou and Zuo, 1990; Kuliev et al., 2004; Melikuziev et al., 2013; Chen H. J. et al., 2012; Suo et al., 2004
44	Methyl gallate	C_8_H_8_O_5_	*R. crenulata*, *R. rosea*, *R. kirilowii*, *R. wallichiana*, *R. sachalinensis*	Wang C. H. et al., 2020; Zhou et al., 2015; Yang et al., 2011a; Zhao et al., 2016; Ding et al., 2018
45	Ethyl gallate	C_9_H_10_O_5_	*R. crenulata*, *R. rosea*, *R. fastigiata*, *R. wallichiana*, *R. tangutica*, *R. sachalinensis*, *R. quadrifida*, *R. sacra*, *R. himalensis*, *R. sexifolia*	Ou et al., 2020; Nan et al., 2018; Qiu et al., 1991; Liu et al., 2024b; Han et al., 2016; Wang H. L. et al., 2006; Chen et al., 1991; Qiu et al., 1989; Suo et al., 2004; Li, 2015
46	Ellagic acid	C_14_H_6_O_8_	*R. crenulata*	Du and Xie (1994)
47	Gallic acid 4-*O*-*β*-D-glucopyranoside	C_13_H_16_O_10_	*R. sacra*, *R. recticaulis*	Olennikov et al., 2019; Fan et al., 1999
48	1-*O*-Galloyl-*β*-D-glucopyranoside	C_13_H_16_O_10_	*R. rosea*	Iwashina et al. (2024)
49	6-*O*-Galloyl-*β*-D-glucopyranoside	C_13_H_16_O_10_	*R. rosea*	Iwashina et al. (2024)
50	1,6-Di-*O*-galloyl-*β*-D-glucopyranoside	C_20_H_20_O_14_	*R. rosea*	Olennikov et al. (2020)
51	2,6-Di-*O*-galloyl-*β*-D-glucopyranoside	C_20_H_20_O_14_	*R. rosea*	Iwashina et al. (2024)
52	1,2,3-Tri-*O*-galloyl-*β*-D-glucopyranoside	C_27_H_24_O_18_	*R. rosea*	Iwashina et al. (2024)
53	1,2,6-Tri-*O*-galloyl-*β*-D-glucopyranoside	C_27_H_24_O_18_	*R. rosea*	Lee et al. (2016)
54	1,3,6-Tri-*O*-galloyl-*β*-D-glucopyranoside	C_27_H_24_O_18_	*R. crenulata*, *R. rosea*	Olennikov et al., 2020; Luo et al., 2024
55	1,2,3,4-Tetra-*O*-galloyl-*β*-D-glucopyranoside	C_34_H_28_O_22_	*R. rosea*	Iwashina et al. (2024)
56	1,2,3,6-Tetra-*O*-galloyl-*β*-D-glucopyranoside	C_34_H_28_O_22_	*R. crenulata*, *R. rosea*, *R. sachalinensis*, *R. yunnanensis*	Lee et al., 2016; Fan et al., 2001; Luo et al., 2024; He and Jitian, 1995
57	2,3,4,6-Tetra-*O*-galloyl-*β*-D-glucopyranoside	C_34_H_28_O_22_	*R. rosea*	Iwashina et al. (2024)
58	1,3-Di-*p*-digalloyl-2,4-digalloyl-*β*-D-glucopyranoside	C_48_H_36_O_30_	*R. rosea*	Iwashina et al. (2024)
59	1,2,3,6-Tetra-*O*-galloyl-4-*O*-(*p*-hydroxybenzoyl)-*β*-D-glucopyranoside	C_41_H_32_O_24_	*R. rosea*	Lee et al. (2016)
60	1,2,3,4,6-Penta-*O*-galloyl-*β*-D-glucopyranose	C_41_H_32_O_26_	*R. crenulata*, *R. rosea*, *R. sachalinensis*, *R. yunnanensis*, *R. henryi*	Wang C. H. et al., 2020; Fan et al., 2001; Chen et al., 2024; Lou and Zuo, 1990; He and Jitian, 1995
61	3-*O*-Methylgallic acid	C_8_H_8_O_5_	*R. crenulata*, *R. wallichiana*, *R. bupleuroides*	Zhao et al., 2016; Dong et al., 2009; Yang et al., 2012a
62	Syringic acid	C_9_H_10_O_5_	*R. rosea*, *R. wallichiana*, *R. bupleuroides*	Song et al., 2018; Dong et al., 2007; Wglarz et al., 2008
63	Glucosyringic acid	C_15_H_20_O_10_	*R. crenulata*	Yang et al. (2012a)
64	Erigeside C	C_15_H_20_O_10_	*R. bupleuroides*	Dong et al. (2007)
65	7-*O*-Syringic-*β*-D-sedoheptulose	C_16_H_22_O_11_	*R. wallichiana*	Chai (2015)
66	Creoside V	C_18_H_24_O_11_	*R. rosea*	Liu et al. (2024b)
67	4,6-Dihydroxy-2-*O*-(*β*-D-glucopyranosyl)acetophenone	C_14_H_18_O_9_	*R. sachalinensis*	Ding et al. (2018)
68	Domesticoside	C_15_H_20_O_9_	*R. tangutica*	Zhang et al. (2010)
69	2,4-Dihydroxy-6-methoxyphenylethanone	C_9_H_10_O_4_	*R. sachalinensis*	Ding et al. (2018)
70	Rodiolinozide	C_15_H_20_O_9_	*R. kirilowii*	Zuo et al. (2007)
71	2,6-Dimethoxy-4-hydroxyacetophenone	C_10_H_12_O_4_	*R. sachalinensis*	Ding et al. (2018)
72	2,6-Dimethoxyacetophenone 4-*O*-*β*-D-glucopyranoside	C_16_H_22_O_9_	*R. crenulata*, *R. wallichiana*, *R. sachalinensis*, *R. coccinea*, *R. recticaulis*	Olennikov et al., 2019; Chai et al., 2015; Qu et al., 2020; Ding et al., 2018; Zong et al., 1991
73	Mallonanoside A	C_14_H_18_O_10_	*R. rosea*	Liu et al. (2024b)
74	Polydatin	C_20_H_22_O_8_	*R. rosea*	Liu et al. (2024b)
75	(*E*)-2,3,5,4′-Tetra-hydroxy-stilbene-2-*O-β*-D-glucopyranoside	C_20_H_22_O_9_	*R. rosea*	Liu et al. (2024b)
76	Oxyresveratrol	C_14_H_12_O_4_	*R. rosea*	Liu et al. (2024b)
77	2-(3,4-Dihydroxyphenyl)-1,3-benzodioxole-5-carboxaldehyde	C_14_H_10_O_5_	*R. rosea*	Liu et al. (2024b)
78	*α*-Tocopherol	C_29_H_50_O_2_	*R. rosea*, *R. imbricata*	Zhang et al., 2022; Tayade et al., 2013
79	*α*-Tocotrienol	C_29_H_44_O_2_	*R. rosea*	Zhang et al. (2022)
80	*β*-Tocotrienol	C_28_H_42_O_2_	*R. rosea*	Zhang et al. (2022)
81	*γ*-Tocopherol	C_28_H_48_O_2_	*R. rosea*	Zhang et al. (2022)
82	*γ*-Tocotrienol	C_28_H_42_O_2_	*R. rosea*	Zhang et al. (2022)
83	*δ*-Tocotrienol	C_27_H_40_O_2_	*R. rosea*	Zhang et al. (2022)
84	Stearic acid	C_18_H_36_O_2_	*R. crenulata*	Li et al. (2012)
85	Linoleic acid	C_18_H_32_O_2_	*R. rosea*	Michels et al. (2020)
86	Mollisinol C	C_18_H_34_O_4_	*R. rosea*	Liu et al. (2024b)
87	Cerotic acid	C_26_H_52_O_2_	*R. crenulata*, *R. yunnanensis*, *R. purpureoviridis*, *R. pamiroalaica*	Ma et al., 1995; Li et al., 2012; Guo et al., 1993; Liu et al., 2000
88	Hexacosanyl cerotate	C_52_H_104_O_2_	*R. yunnanensis*	Guo et al. (1993)
89	Octacosyl cerotate	C_54_H_108_O_2_	*R. henryi*	Lou and Zuo (1991)
90	Glycerin-1-cerotyl-2-linoleate	C_47_H_88_O_5_	*R. henryi*	Lou and Zuo (1991)
91	Octacosanoic acid	C_28_H_56_O_2_	*R. crenulata*	Zhao (2015)
92	Dotriacontanoic acid	C_32_H_64_O_2_	*R. rosea*	Liu et al. (2024b)
93	Hexatriacontanoic acid	C_36_H_72_O_2_	*R. henryi*	Lou and Zuo (1991)
94	2-Furoic acid	C_5_H_4_O_3_	*R. wallichiana*	Zhao (2016)
95	Ethyl benzoate	C_9_H_10_O_2_	*R. crenulata*	Zhao (2015)
96	Dibutyl phthalate	C_16_H_22_O_4_	*R. rosea*, *R. sachalinensis*	Ma, 2006; Ding et al., 2018
97	Fastigitin A	C_13_H_24_O_8_	*R. fastigiata*	Yang et al. (2002)
98	Succinic acid	C_4_H_6_O_4_	*R. henryi*	Lou and Zuo (1990)
99	Suberic acid	C_8_H_14_O_4_	*R. sacra*	Fan et al. (1999)
100	Citric acid	C_6_H_8_O_7_	*R. crenulata*, *R. kirilowii*, *R. fastigiata*, *R. wallichiana*, *R. yunnanensis*, *R. sacra*, *R. himalensis*, *R. tieghemii*	Ma et al., 2022; Zhu et al., 2024

### 3.6 Cyanogenic glycosides

**FIGURE 6:**
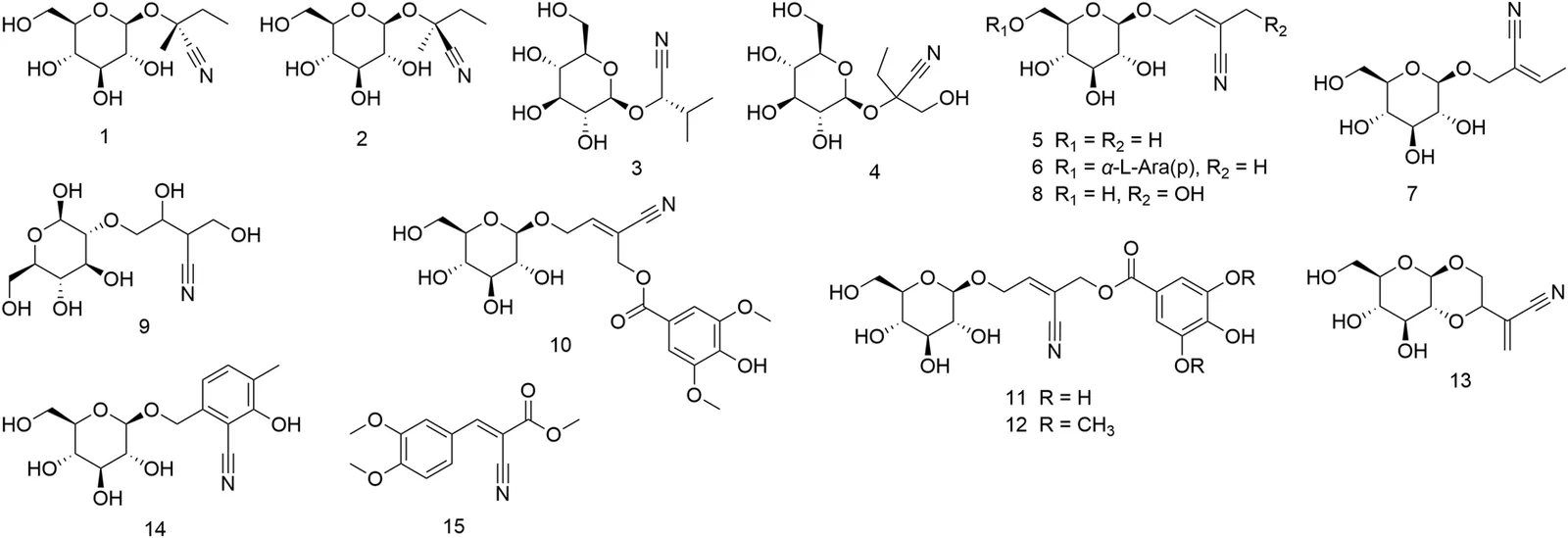
Structures of cyanogenic glycosides.

Cyanogenic glycosides, defined by their -CN functional group that enables toxic HCN release upon enzymatic or acid hydrolysis, represent a chemically distinct class of secondary metabolites in *Rhodiola* species. This review systematically documents 15 such compounds from phytochemical investigations of the genus. Lotaustralin exemplifies the prototypical member of this class. The details of these compounds are presented in Table 7 and Figure 6.

**TABLE 7:** Cyanogenic glycosides from *Rhodiola* species.

No.	Compounds	Molecular formula	Source of species	References
1	(*R*)-Lotaustralin	C_11_H_19_NO_6_	*R. rosea*, *R. kirilowii*, *R. wallichiana*, *R. tangutica*, *R. sachalinensis*, *R. quadrifida*, *R. sacra*, *R. heterodonta*, *R. gelida*, *R. coccinea*, *R. linearifolia*, *R. pamiroalaica*, *R. litwinowii*, *R. semenovii*, *R. subopposita*, *R. junggarica*, *R. recticaulis*	Wang and Ruan, 2005; Olennikov et al., 2019; Kang et al., 1998; Zhang et al., 2010; Nakamura et al., 2007; Zhao et al., 2016; Masi et al., 2023; Yousef et al., 2006; Yoshikawa et al., 1997; Wiedenfeld et al., 2007b
2	Epilotaustralin	C_11_H_19_NO_6_	*R. sachalinensis*	Hou et al. (2009)
3	Heterodendrin	C_11_H_19_NO_6_	*R. sachalinensis*, *R. sacra*, *R. henryi*	Nakamura et al., 2007; Lou and Zuo, 1990; Yoshikawa et al., 1997
4	Sachaloside V	C_11_H_19_NO_7_	*R. sachalinensis*	Nakamura et al. (2007)
5	Rhodiocyanoside A	C_11_H_17_NO_6_	*R. crenulata*, *R. rosea*, *R. kirilowii*, *R. wallichiana*, *R. sachalinensis*, *R. quadrifida*, *R. sacra*, *R. heterodonta*, *R. gelida*, *R. semenovii*, *R. recticaulis*	Olennikov et al., 2019; Nakamura et al., 2007; Wang et al., 2015; Yang et al., 2012a; Yoshikawa et al., 1996; Masi et al., 2023; Yousef et al., 2006; Yoshikawa et al., 1997; Wiedenfeld et al., 2007b
6	Rhodiocyanoside F	C_16_H_25_NO_10_	*R. sacra*	Daikonya and Kitanaka (2011)
7	Rhodiocyanoside D	C_11_H_17_NO_6_	*R. crenulata*, *R. sacra*	Yang et al., 2013; Yoshikawa et al., 1997
8	Sarmentosin	C_11_H_17_NO_7_	*R. crenulata*, *R. sachalinensis*, *R. sacra*, *R. gelida*, *R. recticaulis*	Olennikov et al., 2019; Fan et al., 1999; Yang et al., 2012a; Hou et al., 2009
9	4-(*β*-D-glucopyranosyloxy)-3-hydroxy-2-(hydroxymethyl)-butanenitrile	C_11_H_19_NO_8_	*R. kirilowii*	Yang et al. (2011b)
10	Rhobupcyanoside A	C_20_H_25_NO_11_	*R. wallichiana*, *R. bupleuroides*	Chai et al., 2015; Dong et al., 2009
11	Rhodiocyanoside B	C_18_H_21_NO_11_	*R. quadrifida*, *R. sacra*	Daikonya and Kitanaka, 2011; Yoshikawa et al., 1996
12	*trans*-Rhobupcyanoside A	C_20_H_25_NO_11_	*R. wallichiana*	Chai (2015)
13	Rhobupcyanoside B	C_11_H_15_NO_6_	*R. bupleuroides*	Wang H. et al. (2016)
14	Crenulatanoside A	C_15_H_19_NO_7_	*R. crenulata*	Yang et al. (2012a)
15	Methyl (*E*)-2-cyano-3-(3,4-dimethoxyphenyl)prop-2-enoate	C_13_H_13_NO_4_	*R. crenulata*	Cao and Zhang (2022)

### 3.7 Steroids

**FIGURE 7:**
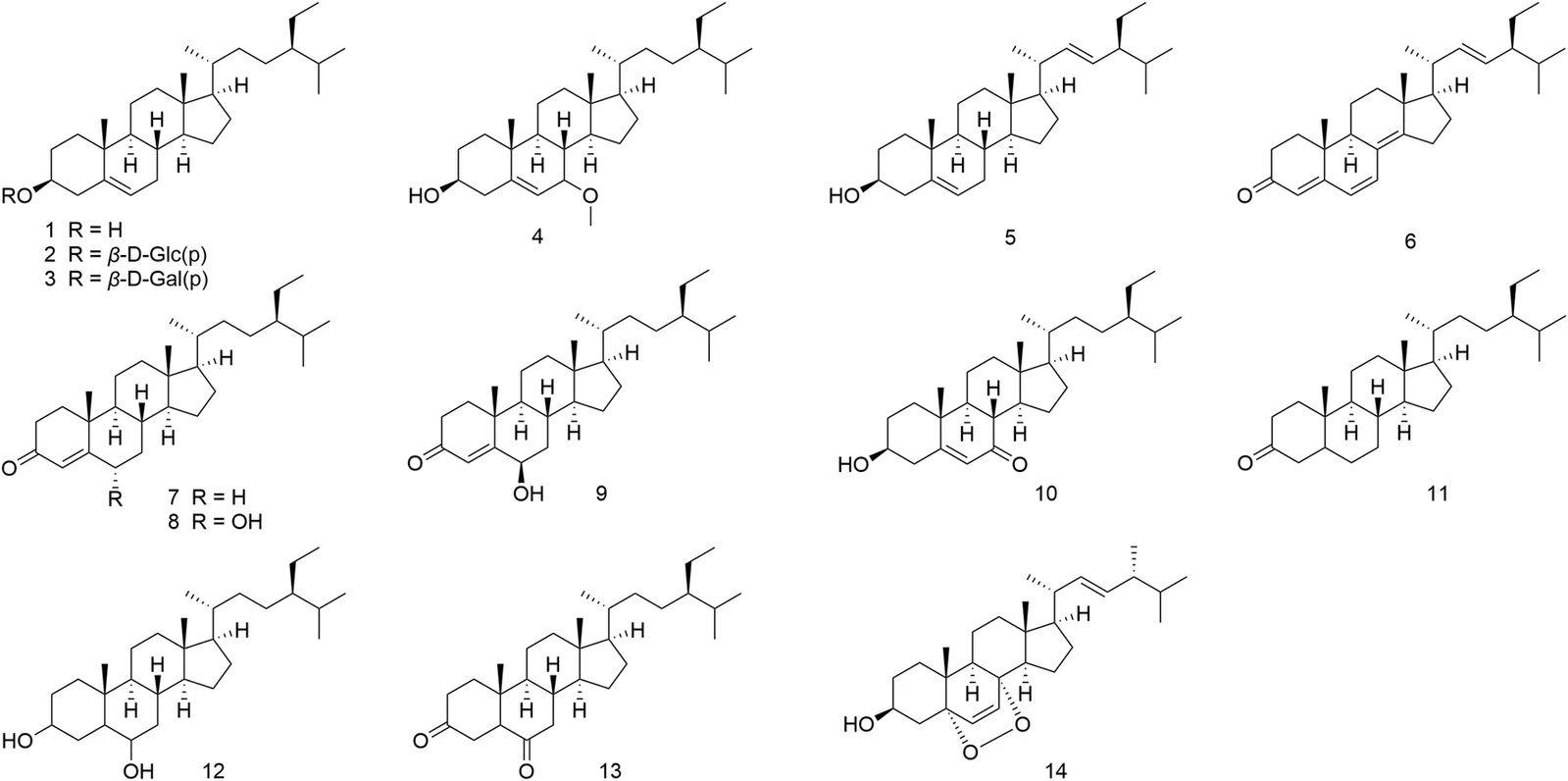
Structures of steroids.

Steroid diversity in the genus *Rhodiola* is limited, with only 14 compounds isolated so far. *β*-Sitosterol and daucosterol are the most representative, having been identified in over 10 *Rhodiola* species. The details of these compounds are presented in Table 8 and Figure 7.

**TABLE 8:** Steroids from *Rhodiola* species.

No.	Compounds	Molecular formula	Source of species	References
1	*β*-Sitosterol	C_29_H_50_O	*R. crenulata*, *R. rosea*, *R. kirilowii*, *R. fastigiata*, *R. wallichiana*, *R. sachalinensis*, *R. yunnanensis*, *R. dumulosa*, *R. sacra*, *R. bupleuroides*, *R. henryi*, *R. himalensis*, *R. atuntsuensis*, *R. linearifolia*, *R. purpureoviridis*, *R. pamiroalaica*	Yang et al., 2002; Qiu et al., 1991; Chen et al., 1999; Ma et al., 1995; Lee et al., 2016; Huang Y. J. et al., 2008; Li et al., 2012; Guo et al., 1993; Lou and Zuo, 1991; Kurlin and Zapesochnaya, 1986; Luo et al., 2005; Zhang, 2021; Liu et al., 2000; Dong et al., 2007; Qiu et al., 1989; Li et al., 1993
2	Daucosterol	C_35_H_60_O_6_	*R. crenulata*, *R. rosea*, *R. kirilowii*, *R. fastigiata*, *R. wallichiana*, *R. sachalinensis*, *R. yunnanensis*, *R. dumulosa*, *R. sacra*, *R. henryi*, *R. himalensis*, *R. linearifolia*, *R. purpureoviridis*	Yang et al., 2002; Qiu et al., 1991; Ma et al., 1995; Song et al., 2003; Qu et al., 2020; Michels et al., 2020; Guo et al., 1993; Kurlin and Zapesochnaya, 1986; Zhang, 2021; Wang J. X. et al., 2006; Lou and Zuo, 1990; Qiu et al., 1989; Peng et al., 1994
3	*β*-Sitosterol 3-*O*-*β*-D-galactopyranoside	C_35_H_60_O_6_	*R. fastigiata*	Chen et al. (1991)
4	Schleicheol 1	C_30_H_52_O_2_	*R. fastigiata*	Gou et al. (2024)
5	Stigmasterol	C_29_H_48_O	*R. kirilowii*	Yang et al. (2011a)
6	Stigmast-4,6,8(14),22-tetraen-3-one	C_29_H_42_O	*R. fastigiata*	Gou et al. (2024)
7	*β*-Sitostenone	C_29_H_48_O	*R. rosea*, *R. fastigiata*, *R. wallichiana*	Zhao, 2016; Michels et al., 2020; Gou et al., 2024
8	6*α*-Hydroxystigmast-4-en-3-one	C_29_H_48_O_2_	*R. fastigiata*	Gou et al. (2024)
9	6*β*-Hydroxystigmast-4-en-3-one	C_29_H_48_O_2_	*R. fastigiata*	Gou et al. (2024)
10	3*β*-Hydroxyporiferast-5-en-7-one	C_29_H_48_O_2_	*R. fastigiata*	Gou et al. (2024)
11	Stigmastan-3-one	C_29_H_50_O	*R. fastigiata*	Gou et al. (2024)
12	Stigmastane-3,6-diol	C_29_H_52_O_2_	*R. fastigiata*	Gou et al. (2024)
13	Stigmastane-3,6-dione	C_29_H_48_O_2_	*R. wallichiana*	Zhao (2016)
14	Ergosterol endoperoxide	C_28_H_44_O_3_	*R. fastigiata*	Gou et al. (2024)

### 3.8 Other compounds

**FIGURE 8:**
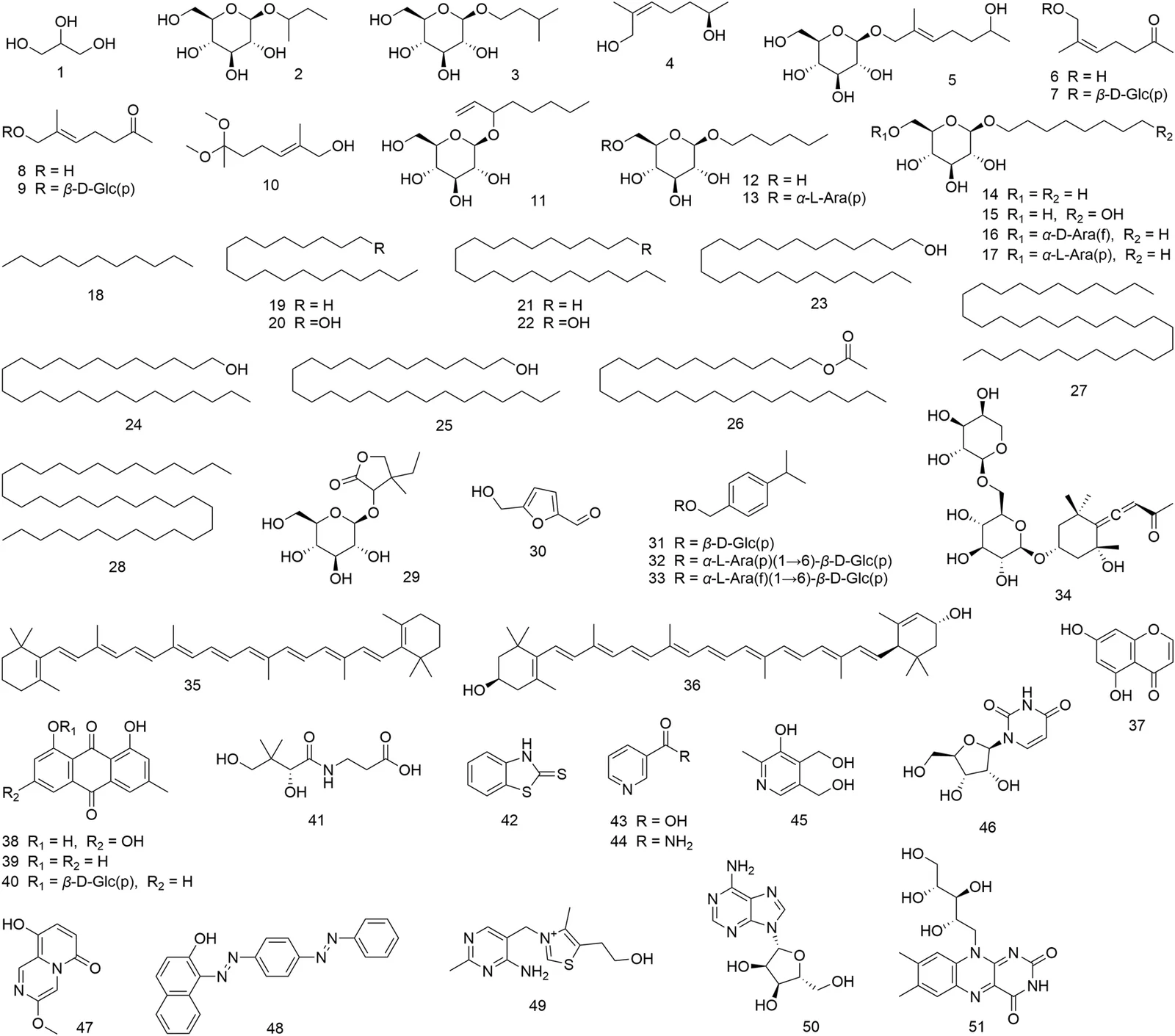
Structures of other compounds.

Beyond the aforementioned major phytochemical classes, *Rhodiola* species also produce alkanols, olefinic alcohols, alkanes, anthraquinones, and several other unclassified metabolites, further contributing to their chemical diversity. The details of these compounds are presented in Table 9 and Figure 8.

**TABLE 9:** Other compounds from *Rhodiola* species.

No.	Compounds	Molecular formula	Source of species	References
1	Glycerin	C_3_H_8_O_3_	*R. wallichiana*	Chai (2015)
2	sec-Butyl-*O*-*β*-D-glucopyranoside	C_10_H_20_O_6_	*R. wallichiana*	Zhao et al. (2016)
3	3-Methyl-1-butanol *β*-D-glucopyranoside	C_11_H_22_O_6_	*R. rosea*	Ma (2006)
4	(*R*,*Z*)-2-Methyl-2-hepten-1,6-diol	C_8_H_16_O_2_	*R. rosea*	Lee et al. (2016)
5	Creoside II	C_14_H_26_O_7_	*R. crenulata*	Nakamura et al. (2008)
6	(*Z*)-2-Methyl-6-oxo-2-hepten-1-ol	C_8_H_14_O_2_	*R. rosea*	Lee et al. (2016)
7	(*Z*)-Creoside I	C_14_H_24_O_7_	*R. crenulata*, *R. rosea*	Lee et al., 2016; Nakamura et al., 2008
8	(*E*)-2-Methyl-6-oxo-2-hepten-1-ol	C_8_H_14_O_2_	*R. rosea*	Kim et al. (2017)
9	(*E*)-Creoside I	C_14_H_24_O_7_	*R. rosea*	Lee et al. (2016)
10	(*E*)-2-Methyl-6,6-dimethoxy-2-hepten-1-ol	C_10_H_20_O_3_	*R. rosea*	Kim et al. (2017)
11	1-Octen-3-ol 3-*O-β*-D-glucopyranoside	C_14_H_26_O_6_	*R. fastigiata*	Yang et al. (2002)
12	Hexyl *β*-D-glucopyranoside	C_12_H_24_O_6_	*R. crenulata*, *R. rosea*, *R. kirilowii*, *R. quadrifida*	Nakamura et al., 2008; Liu et al., 2024b; Huang Y. J. et al., 2008; Yoshikawa et al., 1996
13	Creoside IV	C_17_H_32_O_10_	*R. crenulata*, *R. rosea*	Nakamura et al., 2008; Liu et al., 2024b
14	Octyl *β*-D-glucopyranoside	C_14_H_28_O_6_	*R. sachalinensis*	Nakamura et al. (2007)
15	8-Hydroxyoctyl-*β*-D-glucopyranoside	C_14_H_28_O_7_	*R. rosea*	Liu et al. (2024b)
16	1-Octyl-*α*-D-arabinofuranosyl(1→6)-*β*-D-glucopyranoside	C_19_H_36_O_10_	*R. sachalinensis*	Nakamura et al. (2007)
17	Rhodiooctanoside	C_19_H_36_O_10_	*R. crenulata*, *R. kirilowii*, *R. quadrifida*, *R. sacra*	Nakamura et al., 2008; Huang Y. J. et al., 2008; Yoshikawa et al., 1996; Yoshikawa et al., 1997
18	*n*-Undecane	C_11_H_24_	*R. sachalinensis*	Ding et al. (2018)
19	*n*-Nonadecane	C_19_H_40_	*R. crenulata*	Li et al. (2012)
20	1-Nonadecanol	C_19_H_40_O	*R. purpureoviridis*	Ma et al. (1995)
21	*n*-Docosane	C_22_H_46_	*R. pamiroalaica*	Liu et al. (2000)
22	1-Docosanol	C_22_H_46_O	*R. fastigiata*, *R. purpureoviridis*	Ma et al., 1995; Gou et al., 2024
23	1-Tetracosanol	C_24_H_50_O	*R. fastigiata*, *R. wallichiana*	Zhang, 2021; Gou et al., 2024
24	1-Hexacosanol	C_26_H_54_O	*R. crenulata*, *R. yunnanensis*	Li et al., 2012; Guo et al., 1993
25	1-Heptacosanol	C_27_H_56_O	*R. wallichiana*, *R. purpureoviridis*	Ma et al., 1995; Zhang, 2021
26	Octacosyl acetate	C_30_H_60_O_2_	*R. yunnanensis*	Guo et al. (1993)
27	*n*-Pentatriacontane	C_35_H_72_	*R. sachalinensis*	Ding et al. (2018)
28	*n*-Heptatriacontane	C_37_H_76_	*R. henryi*	Lou and Zuo (1991)
29	Algidin	C_13_H_22_O_8_	*R. tangutica*	Pangarova et al. (1975)
30	5-Hydroxymethylfurfural	C_6_H_6_O_3_	*R. wallichiana*	Ou et al. (2020)
31	Cuminyl *β*-D-glucopyranoside	C_16_H_24_O_6_	*R. rosea*	Tang et al. (2020)
32	Cuminyl *α*-L-arabinopyranosyl(1→6)-*β*-D-glucopyranoside	C_21_H_32_O_10_	*R. rosea*	Tang et al. (2020)
33	Cuminyl *α*-L-arabinofuranosyl(1→6)-*β*-D-glucopyranoside	C_21_H_32_O_10_	*R. rosea*	Tang et al. (2020)
34	Crenulatanoside B	C_24_H_38_O_12_	*R. crenulata*	Yang et al. (2012b)
35	*β*-Carotene	C_40_H_56_	*R. rosea*	Zhang et al. (2022)
36	Lutein	C_40_H_56_O_2_	*R. rosea*	Zhang et al. (2022)
37	5,7-Dihydroxychromone	C_9_H_6_O_4_	*R. wallichiana*	Zhao (2016)
38	Emodin	C_15_H_10_O_5_	*R. sachalinensis*	Ding et al. (2018)
39	Chrysophanol	C_15_H_10_O_4_	*R. yunnanensis*	Guo et al. (1993)
40	Chrysophanol 8-*O-β*-D-glucopyranoside	C_21_H_20_O_9_	*R. crenulata*, *R. dumulosa*	Wang J. X. et al., 2006; Sun et al., 2020
41	D-Pantothenic acid	C_9_H_17_NO_5_	*R. imbricata*	Tayade et al. (2013)
42	2(3H)-Benzothiazolethione	C_7_H_5_NS_2_	*R. sachalinensis*	Han et al. (2011)
43	Nicotinic acid	C_6_H_5_NO_2_	*R. imbricata*	Tayade et al. (2013)
44	Nicotinamide	C_6_H_6_N_2_O	*R. imbricata*	Tayade et al. (2013)
45	Pyridoxine	C_8_H_11_NO_3_	*R. imbricata*	Tayade et al. (2013)
46	Uridine	C_9_H_12_O_6_N_2_	*R. crenulata*	Qu et al. (2020)
47	Xylogranatinin	C_9_H_8_N_2_O_3_	*R. crenulata*	Liu et al. (2024a)
48	Sudan III	C_22_H_16_N_4_O	*R. sachalinensis*	Ding et al. (2018)
49	Thiamine	C_12_H_17_N_4_OS^+^	*R. imbricata*	Tayade et al. (2013)
50	Adenosine	C_10_H_13_N_5_O_4_	*R. crenulata*	Ren et al. (2021)
51	Riboflavin	C_17_H_20_N_4_O_6_	*R. imbricata*	Tayade et al. (2013)

In summary, as the first step toward discovering chemical markers for *Rhodiola* species identification, this comprehensive phytochemical review clarifies the current research progress on the chemical constituents in the genus *Rhodiola*. It reveals an exceptionally rich and diverse chemical profile, with over 500 compounds isolated and identified from the genus into eight structural classes. The remarkable chemical diversity poses one of the notable challenges for marker discovery, as the complex matrix—especially a large number of compounds with similar structures or polarities—increases the technical difficulty of achieving effective chromatographic separation of potential marker compounds.

Critically, the compiled data provides a clear baseline for the preliminary qualitative understanding of the phytochemical composition across different *Rhodiola* species. On the one hand, the compiled data, as summarized in Tables 2–9, underscore the fundamental fact that the vast majority of identified constituents are not exclusive to a single species but shared across multiple ones, reflecting the chemical similarities among closely related species. This constitutes another notable challenge for marker discovery, substantially reducing the likelihood of identifying strictly species-specific compounds. On the other hand, the compiled data reveal clear interspecific chemical differences at the qualitative level, laying an initial foundation for *Rhodiola* species identification based on chemical constituents. Specifically, while most constituents are shared across multiple species, their distribution ranges within the genus *Rhodiola* vary greatly. The interspecific differences therefore lie primarily in the distinct combinations of shared compounds among different species. It should be noted that although a small subset of compounds have so far been isolated or detected only in individual species, they may not necessarily serve as valid species-specific markers for *Rhodiola* species identification, given that their strict interspecific exclusivity remains unverified and their practical applicability in routine detection requires further confirmation.

Meanwhile, the comprehensive compound inventory presented in this review—encompassing chemical names, molecular formulas, chemical structures, and botanical sources of all documented compounds—constitutes an essential chemical constituent database for *Rhodiola* species. As a valuable supplementary outcome of this review, the database serves as a key reference for qualitative chemical analysis of various *Rhodiola* species. It will provide standardized information to support compound identification and facilitate the systematic screening of chemical markers for distinguishing different *Rhodiola* species in future research.

## 4 Discussion

Chemical constituent-based species identification is a crucial yet technically challenging aspect of Chinese herbal medicine identification, especially when authenticating the botanical sources of raw materials used in herbal preparations. The species identification issues associated with *Rhodiola* stand as a prototypical example in this field. Despite inherent difficulties, seeking potential chemical markers for *Rhodiola* species remains imperative to address the practical need for authenticating raw materials in *Rhodiola* preparations, thereby strengthening market supervision and mitigating adulteration risks. Building upon the above review of chemical constituents in the genus *Rhodiola*, the following discussion further puts forward new insights and strategies that offer concrete research direction to tackle current challenges.

### 4.1 Limitations of species-specific compounds for *Rhodiola* species identification

The conventional approach to chemical constituent-based species identification prioritizes seeking species-specific compounds as ideal markers. However, while interspecific differences in chemical profiles do exist (Ma et al., 2021), two fundamental obstacles inherently hinder the discovery of such compounds as reliable chemical markers: (1) pronounced interspecific phytochemical similarities (Li et al., 2019) stemming from close phylogenetic relationships, which make truly species-specific compounds exceedingly rare and greatly reduce the likelihood of finding them; and (2) considerable intraspecific phytochemical variability (Liu et al., 2013) induced by extrinsic factors (e.g., geographic origin, plant age, harvest time, and storage conditions), which means that even a putatively specific compound may be present at low abundance or be absent in some individuals of the species, thus failing to serve as a stable identification marker.

Our review in this paper fully reflects the interspecific similarities mentioned above, demonstrating that most identified compounds are shared across multiple *Rhodiola* species. While interspecific differences are also clearly illustrated in the review—particularly evidenced by certain compounds reported so far in only a single species—none of these have been formally designated as identification markers for their respective species in published literature. This situation is likely attributed to two primary reasons: first, the species specificity and intraspecific stability of these compounds remain unvalidated; second, they may fail to meet key requirements for routine analysis, including (1) effective chromatographic resolution from compounds with similar polarity or structure, (2) commercial availability of reference standards, and (3) sufficient natural abundance to ensure reliable detection—even after extraction or processing. Consequently, it should be recognised that relying solely on the presence/absence of species-specific compounds for *Rhodiola* species identification poses considerable practical challenges.

### 4.2 Content levels/ratios as potential markers for *Rhodiola* species identification

Traditionally, chemical constituents shared among multiple *Rhodiola* species (e.g., salidroside, tyrosol, rhodionin, and gallic acid) have been excluded as diagnostic markers for species identification. Although these constituents are universally recognized as bioactive compounds (Ji et al., 2025; Wang et al., 2026; Choe et al., 2012), their ubiquitous distribution across nearly all chemically investigated *Rhodiola* species (Zhu et al., 2024; Li and Zhang, 2008) limits their utility for species identification in most studies. Instead, their contents are routinely recommended for quality control purposes.

However, a critical oversight persists that the reported significant chemical differences between closely related species in most studies are primarily derived from quantitative comparison of shared phytochemicals rather than qualitative presence/absence (Ma et al., 2021; Liu et al., 2013). In other words, these shared compounds exist across multiple species but exhibit highly variable content levels among different ones. This suggests that we should move beyond the prevailing paradigm of solely pursuing species-specific compounds and strategically incorporate quantitative data into species identification frameworks.

In fact, quantitative approaches have already demonstrated promise in *Rhodiola* species identification. Cinnamic alcohol derivatives (rosavin, rosarin, and rosin, collectively termed “rosavins”) were originally claimed to be unique to *R. rosea* in a few earlier papers. However, with continuous advances in analytical techniques and expanded sampling/species coverage, evidence from subsequent studies has revealed their presence in some congeneric species (e.g., *R. crenulata*, *R. kirilowii*, and *R. sachalinensis*, as reviewed above), albeit at markedly lower concentrations (Shao et al., 2004; Ma et al., 2008). Thus, the diagnostic value of rosavins for *R. rosea* lies not in exclusivity but in their substantially higher content relative to other *Rhodiola* species—a more accurate way to distinguish *R. rosea* than relying on claimed absolute specificity. As noted in the Introduction, this quantitative principle (i.e., quantifying rosavins content) is already utilized to detect adulteration in *R. rosea* preparations (Booker et al., 2016b; Ma et al., 2011). Although current applications of the content are limited to distinguishing *R. rosea* from other related species, this case clearly illustrates the potential of content levels in *Rhodiola* species identification.

Furthermore, content ratios serve as a crucial complementary dimension to absolute content data, meriting focused attention in *Rhodiola* species identification. For a given species, phytochemical contents frequently vary significantly due to factors such as geographic origin, plant age, and harvest time, sometimes resulting in intraspecific variation surpassing interspecific differences. In contrast, the content ratios between certain compounds may remain relatively stable to a large extent, thereby offering enhanced diagnostic reliability for particular species. As early as 2013, Wieland Peschel put forward the preliminary idea of utilizing content ratios for *R. rosea* authentication (Peschel et al., 2013). Although this promising idea remains unexplored and unvalidated in any *Rhodiola* species to date, to the best of our knowledge, filling this gap through systematic investigation and application of these relatively stable ratios may provide a potential solution to the current limitations of chemical constituent-based *Rhodiola* species identification.

### 4.3 Enhancing the feasibility of *Rhodiola* species identification with a hierarchical “chemical characteristics + classification tree” framework

In the previous section, we proposed an upgraded strategy for identifying *Rhodiola* species based on chemical constituents. This approach shifts the research focus of chemical markers from only species-specific compounds to broader chemical characteristics. In this context, this term differs from “features” commonly used in chemometrics, where the latter generally refers to raw or processed analytical data (e.g., peak areas, mass spectrometry fragments). For this work, chemical characteristics are herein defined as encompassing species-specific compounds, content levels, and content ratios. By broadening the research scope of chemical markers, this strategy no longer relies solely on the presence or absence of species-specific compounds for species identification, thereby substantially increasing the probability of discovering valid chemical markers.

Nevertheless, deciphering a single, definitive chemical characteristic unique to each *Rhodiola* species may still be challenging, since, as concluded from our review, the interspecific chemical differences in this genus are mainly manifested as distinct combinations of shared compounds. Given this distribution pattern of chemical constituents among *Rhodiola* species, we propose adopting a hierarchical framework that integrates chemical characteristics within a classification tree for stepwise identification. It first uses highly discriminative chemical characteristics for broad categorization, then applies secondary characteristics to further subdivide each category, and continues until the target species are unambiguously identified.

Building upon the expanded scope of chemical markers outlined above, our proposed framework further overcomes the inherent limitation of the conventional identification paradigm—seeking single species-specific markers for direct one-step species identification. By contrast, this framework employs a multi-step discrimination mode to progressively narrow down the identification scope. Such a design is better aligned with the actual interspecific chemical distribution patterns observed in the genus *Rhodiola*. As a result, the hierarchical “chemical characteristics + classification tree” framework reduces the analytical complexity of deciphering intricate chemical profiles, which in turn enhances the feasibility of chemical constituent-based *Rhodiola* species identification.

### 4.4 An integrated future research plan for *Rhodiola* species identification

To tackle the challenge of identifying *Rhodiola* species via their chemical constituents, the previous section analyzed the limitations of relying solely on species-specific compounds, argued for the potential of using content levels/ratios as complementary markers, and innovatively proposed a hierarchical “chemical characteristics + classification tree” framework to enhance the feasibility of species identification. Despite their great potential, these insights and strategies remain largely theoretical at present and necessitate validation through extensive, systematic data.

There is rich literature on the isolation, identification, and quantification of chemical constituents in *Rhodiola* species, yet the available studies are highly fragmented: different research groups concentrate on distinct *Rhodiola* species, target varying sets of chemical constituents, and employ inconsistent technical parameters (Polumackanycz et al., 2022; Zhu et al., 2024). Consequently, the existing data are scattered and incomplete, failing to support cross-species verifications of species-specific compounds or meaningful comparisons of content levels/ratios. This makes it difficult to integrate them into a coherent dataset suitable for in-depth analysis of the chemical characteristics of various *Rhodiola* species. In other words, achieving the goal of *Rhodiola* species identification based on chemical constituents necessitates systematic and comprehensive research efforts. Herein, we briefly outline our corresponding research plan for *Rhodiola* species identification.

Step 1: review of chemical constituents

This paper has already completed the first and fundamental step in our research plan. It systematically compiles the currently reported chemical constituents from various members of this genus to provide a macroscopic overview of current research progress on *Rhodiola* phytochemistry. The results preliminarily reveal a remarkable chemical diversity in *Rhodiola* species, along with both interspecific chemical similarities and differences from a qualitative perspective. Furthermore, the consolidated phytochemical data herein can serve as a foundational database, which will offer a “targeted screening list” for subsequent qualitative identification of chemical constituents in different *Rhodiola* species via UPLC-MS. This will help circumvent the inaccuracy associated with “blind detection” in compound identification. Thus, this review creates a necessary prerequisite for the subsequent experimental phases of our research project on chemical constituent-based *Rhodiola* species identification.

Step 2: targeted sample collection

A systematic investigation of *Rhodiola* species currently used clinically and available commercially should be conducted to determine the target species for the present study. For each selected species, a sufficient number of samples must be collected, with full consideration given to key influencing factors such as origin, plant age, and harvest time to ensure both representativeness and comprehensiveness of the samples. This step is directly relevant to the credibility and persuasiveness of the final analytical results and conclusions.

Step 3: UPLC-MS-based qualitative profiling

Systematic qualitative analysis of chemical constituents in the target *Rhodiola* species will be performed using ultra-high performance liquid chromatography-mass spectrometry (UPLC-MS). This analysis will utilize the chemical database established in this paper as a reference framework, and further match compounds with reference standards based on characteristic fragment ions and retention times for precise identification. Given that this step aims to comprehensively identify chemical constituents to the greatest extent (to ensure broader coverage of compounds for quantitative analysis in the next step), two critical aspects must be emphasized during the experiment: (1) screening suitable extraction conditions to simultaneously extract multi-class compounds, including phenylalkanoid derivatives, phenylpropanoids, flavonoids, terpenoids, and other classes; (2) optimizing chromatographic conditions (e.g., mobile phase composition, column temperature, gradient elution program) to maximize chromatographic resolution, enabling efficient separation and detection of a larger number of compounds.

Step 4: UPLC-based quantitative analysis

For all compounds from different *Rhodiola* species that have been unambiguously identified through reference standard matching and exhibit satisfactory chromatographic separation, precise quantification will be performed using ultra-performance liquid chromatography (UPLC). This step is designed to maximize the acquisition of accurate quantitative data, providing a robust analytical foundation for subsequent in-depth exploration of *Rhodiola*’s chemical characteristics.

Step 5: multivariate data analysis for marker discovery

Based on the qualitative and quantitative analysis in the previous steps, the data representing three types of chemical information can be obtained: (1) the presence and absence of compounds across samples (reflected by non-zero/zero content values), (2) the content values of individual compounds, and (3) pairwise ratios between the quantified compound contents. Although these three types of data correspond to distinct chemical dimensions, they are all expressed as numerical variables and can therefore be integrated into a single unified dataset to serve as the foundation for subsequent multivariate analysis.

In terms of the analytical strategy for chemical marker discovery, we plan to adopt a stepwise screening workflow. This process begins with the screening of variables with high discriminatory power for species classification (e.g., principal component analysis to achieve data dimensionality reduction or to reveal the basis for natural clustering of different species) (Anowar et al., 2021). From these pre-screened variables, those that function as branching nodes of the classification tree are subsequently identified (e.g., decision tree algorithm to pinpoint the key nodes) (Tang et al., 2025). Finally, based on the actual measured values of these variables, the specific threshold values or value ranges for each branching node to distinguish different species can be further defined. Following this multivariate analytical pipeline, systematic mining and screening of chemical characteristics will be accomplished to establish a hierarchical “chemical characteristics + classification tree” framework. Within this framework, the stepwise differentiation of *Rhodiola* species is guided by three types of chemical characteristics: (1) species-specific compounds, (2) content levels, and (3) content ratios.

By implementing this systematic research plan, we aim to ultimately develop a reliable, practical chemical identification method for *Rhodiola* species. This method will provide the critical technical support needed to fundamentally solve the problem of raw material authentication in *Rhodiola* preparations, helping to combat adulteration, strengthen market supervision and ensure quality for *Rhodiola* products.

## 5 Conclusion

Given the frequent and potential adulteration in commercial *Rhodiola* preparations, developing an effective method to authenticate the species of raw materials is imperative for strengthening market supervision and ensuring product quality. As key identifying characteristics (morphological, microscopic, and genetic) are often degraded or destroyed during processing, a chemical constituent-based approach emerges as the most promising and valuable research avenue, with the core challenge and breakthrough point lying in the discovery of reliable chemical markers for *Rhodiola* species identification. This paper serves as the critical first step in addressing this challenge by comprehensively reviewing the current knowledge about chemical constituents of the genus *Rhodiola*. From a qualitative perspective, the review clarifies both the inherent difficulties and the potential feasibility of discovering such chemical markers: while the remarkable chemical diversity (as evidenced by over 500 compounds classified into eight major groups) and the pronounced interspecific similarities (most compounds are shared across multiple species) collectively complicate marker discovery, the observed interspecific differences (manifested as distinct combinatorial patterns of shared constituents and compounds currently known from single species) provide a promising foundation for developing a chemical constituent-based *Rhodiola* species identification method. Meanwhile, this comprehensive review also results in a chemical database, offering a “targeted screening list” for compound identification across various *Rhodiola* species during our subsequent research phases of marker discovery. In addition, we propose innovative strategies to enhance the feasibility of chemical constituent-based *Rhodiola* species identification: (1) shifting the research focus of chemical markers from solely pursuing species-specific compounds to exploring broader chemical characteristics (encompassing species-specific compounds, content levels, and content ratios); and (2) adopting a hierarchical “chemical characteristics + classification tree” framework for stepwise identification. Finally, we outline a systematic research plan to translate these theoretical strategies into practice, including the completed foundational review of chemical constituents, systematic sampling, UPLC-MS/UPLC-based chemical profiling, and advanced data analysis.

In summary, this paper provides both a comprehensive review of chemical constituents for *Rhodiola* species and a resulting compound database, which together lay a solid chemical foundation for the marker discovery research outlined in this work. The proposed insights, strategies and corresponding research plan collectively reflect a paradigm shift from seeking “single-point breakthroughs” toward implementing an “integrated, hierarchical framework,” offering a novel pathway to address the long-standing challenge of distinguishing closely related species based on chemical constituents. As a vital supplement to existing methodologies for *Rhodiola* species identification, such a method is particularly poised to resolve the difficulty of authenticating raw materials in processed preparations. It is anticipated that the subsequent research built upon this paper will play a pivotal role in combating market adulteration, strengthening market supervision, and ultimately safeguarding the quality of *Rhodiola* products.
